# Targeted Folate-Chitosan Nanoformulations of Quercetin and *Coriandrum sativum* Reprogram Breast Cancer Hallmarks by Silencing Stemness, Cell Cycle, Angiogenic, and Metastatic Networks

**DOI:** 10.3390/ph19081271

**Published:** 2026-08-12

**Authors:** Nariman Nabil, Hussein Sabit, Jawaher Almulhim, Borros Arneth, Shaimaa Abdel-Ghany

**Affiliations:** 1 Department of Medical Biotechnology, College of Biotechnology, Misr University for Science and Technology, P.O. Box 77, Giza 12566, Egypt; nareeman.nabil@must.edu.eg (N.N.); hussein.sabit@must.edu.eg (H.S.); 2 Department of Biological Sciences, King Faisal University, Alahsa 31982, Saudi Arabia; jalmulhim@kfu.edu.sa; 3 Institute of Laboratory Medicine and Pathobiochemistry, Molecular Diagnostics, Hospital of the Universities of Giessen and Marburg (UKGM), Philipps University Marburg, Baldingerstr. 1, 35043 Marburg, Germany; 4 Institute of Laboratory Medicine and Pathobiochemistry, Molecular Diagnostics, Hospital of the Universities of Giessen and Marburg (UKGM), Justus Liebig University Giessen, Feulgenstr 12, 35392 Giessen, Germany; 5 Department of Environmental Biotechnology, College of Biotechnology, Misr University for Science and Technology, P.O. Box 77, Giza 12566, Egypt; shaimaa.ibraheem@must.edu.eg

**Keywords:** chitosan nanoparticles, folate targeting, quercetin, *Coriandrum sativum*, breast cancer, apoptosis induction, angiogenesis inhibition

## Abstract

**Background/Objectives**: This study engineered and evaluated a targeted, folate-functionalized chitosan nanoparticle (CS-FA NP) delivery system to enhance the therapeutic efficacy of standard quercetin and *Coriandrum sativum* seed extract against breast cancer. **Methods**: Phytochemical profiling confirmed a 14% crude yield for the methanolic extract, with gas chromatography–mass spectrometry (GC-MS) and high-performance liquid chromatography (HPLC) identifying quercetin as the principal bioactive agent. The synthesized CS-FA NPs exhibited a core size of 7–20 nm, an average hydrodynamic diameter of 150–160 nm, a stable zeta potential of −55 mV, and high encapsulation efficiencies (87.2% for quercetin and 80.5% for coriander). Kinetic assessments confirmed a biphasic, diffusion-controlled release matching Higuchi matrix kinetics. Anticancer activity was evaluated in vitro using MTT cytotoxicity, Annexin V-FITC/PI apoptosis analysis, RT-qPCR, and ex vivo rat aortic ring assays, followed by validation in a syngeneic 4T1 mammary tumor mouse model. **Results**: In vitro, folate-receptor-targeted quercetin nanoparticles (T4) demonstrated superior, selective cytotoxicity, particularly against triple-negative MDA-MB-231 cells, while sparing normal fibroblasts. Annexin V-FITC/PI apoptosis profiling and ex vivo aortic ring assays revealed profound, cell-line-dependent programmed cell death and up to 90% inhibition of microvessel sprout outgrowth. Mechanistically, RT-qPCR verified that nano-formulations induced complete transcriptional silencing of NANOG, MMP-1, VEGFA, TSPAN8, TWIST, EMMPRIN, and CDK1, alongside marked upregulation of P27^KIP1^ and P21^CIP1^. In vivo, these nano-formulations successfully improved tumor-associated pathological features, reduced aggressive tumor spindle-cell proliferation, and suppressed elevated serum CA15-3 and arginase biomarkers. **Conclusions**: Folate-functionalized chitosan nano-formulations significantly enhanced the anticancer efficacy of quercetin and *Coriandrum sativum* seed extract through improved targeted delivery, potent antiproliferative, anti-angiogenic, and pro-apoptotic activities, together with favorable modulation of multiple molecular pathways associated with breast cancer progression. These findings support their potential as promising targeted nanotherapeutic strategies for breast cancer treatment.

## 1. Introduction

Breast cancer remains a major global clinical challenge due to its intricate genomic heterogeneity, sub-clonal evolutionary shifts, and the rapid development of resistance to conventional chemotherapies. While preventive strategies increasingly emphasize the protective modulation of cellular microenvironments through antioxidants, vitamins, and customized dietary probiotics [1], treating established malignancies requires targeted molecular interventions.

Breast cancer is a highly heterogeneous disease comprising several molecular subtypes, including luminal A, luminal B, HER2-positive, and triple-negative breast cancer (TNBC) [2]. Each subtype exhibits distinct molecular characteristics, clinical behavior, and therapeutic responses. Among these, TNBC is considered one of the most aggressive forms because it lacks estrogen receptor (ER), progesterone receptor (PR), and human epidermal growth factor receptor 2 (HER2), which limits the availability of targeted therapies [3]. As a result, patients with TNBC often experience rapid disease progression, early recurrence, distant metastasis, and poor overall prognosis. Although conventional treatments such as surgery, chemotherapy, radiotherapy, and targeted therapies have improved survival, their effectiveness is constrained by systemic toxicity, multidrug resistance, poor tumor selectivity, and treatment-related adverse effects. Therefore, developing targeted therapeutic strategies that can selectively deliver anticancer agents to tumor cells while minimizing harm to healthy tissues has become a key objective in breast cancer research.

Folate receptor-α (FRα) is overexpressed in a range of epithelial malignancies, including several breast cancer subtypes, whereas its expression in most normal tissues is relatively low [4]. This differential expression profile has established folic acid as an attractive targeting ligand for nanoparticle-based drug delivery, enabling receptor-mediated endocytosis and increasing intracellular drug accumulation in tumor cells. Concurrently, naturally derived phytochemicals have gained substantial attention as potential anticancer agents owing to their capacity to modulate multiple signaling pathways involved in cell proliferation, apoptosis, angiogenesis, metastasis, and oxidative stress [5]. However, their poor aqueous solubility, limited bioavailability, and rapid systemic clearance markedly constrain their therapeutic effectiveness. Consequently, folate-targeted nanocarriers encapsulating bioactive phytochemicals have emerged as a promising strategy to enhance selective drug delivery, improve therapeutic efficacy, and minimize systemic toxicity in cancer therapy.

Dietary nutraceuticals have transitioned from simple supplements to highly capable chemotherapeutic adjuvants due to their multi-targeted modulation of aberrant signaling networks [6]. Among these bioactive compounds, naturally occurring flavonols are prominent options for treating breast and gynecological malignancies because they interact directly with multiple mutated oncogenic targets [7].

For example, structural variants like dihydroquercetin exhibit potent anti-proliferative activities by inducing cell cycle arrest and modulating cellular oxidation state pathways [8]. Investigating these plant-derived compounds reveals complex mechanical networks that govern cell survival, migration, and apoptosis.

The therapeutic mechanism of the flagship flavanol quercetin relies on its ability to systematically dismantle cell survival signaling. Systematic cell-line evaluations show that quercetin safely triggers programmed cell death pathways in both hormone-responsive MCF-7 and highly aggressive, triple-negative MDA-MB-231 human breast cancer models [9]. This cytotoxic response is often enhanced when quercetin is combined with other bioflavonoids like chrysin, which dynamically alters cell cycle progression, induces G2/M phase arrest, and shifts the balance of pro- and anti-apoptotic proteins [10].

Furthermore, quercetin isolated from wild botanical matrices like *Acalypha indica* maintains strong, reproducible cytotoxic profiles against these standard cell lines, highlighting its stability and natural potency [11]. Mechanistically, this compound halts tumor growth by targeting the core components of the Akt/mTOR/PTEN signaling axis, suppressing downstream protein synthesis and survival signals [12].

Beyond standard translational regulation, quercetin prevents metastasis by disrupting post-transcriptional RNA-binding networks. It interferes directly with the human antigen R (HuR) protein, suppressing HuR-driven progression, cell invasion, and the migration of triple-negative breast cancer cells [13].

Despite its significant molecular potency, translating free quercetin into clinical applications is limited by its poor aqueous solubility, rapid systemic clearance, and low bioavailability. Nanotechnology provides a solution to these issues, transforming quercetin into a reliable therapeutic option by embedding it within specialized nanocarriers to enhance target delivery [14].

Modern nanoparticle architectures are designed to co-deliver nucleic acids alongside conventional anti-cancer drugs, offering a dual-action mechanism to overcome multi-drug resistance [15]. Combining these advanced nanotechnological delivery systems with potent phytochemicals forms a comprehensive strategy to bypass efflux transporters and reverse chemoresistance [16].

Recent efforts to improve the circulation time and tumor selectivity of folate-targeted agents have included conjugating small-molecule drug conjugates (SMDCs) such as EC140 to the Fc region of antibodies at multiple sites. This approach produced a conjugate with a drug-to-protein ratio of approximately 4, retained FcRn recycling, extended plasma half-life to ~28 h in mice, and achieved near-complete regression of folate-receptor-positive KB tumors at modest doses [17]. While these protein-based conjugates demonstrate the power of prolonged systemic exposure combined with folate-receptor engagement, they still rely on a single cytotoxic payload and require sophisticated conjugation chemistry.

Solid lipid nanoparticle (SLN) matrices have proven highly effective for co-encapsulation strategies. For instance, combining the chemotherapeutic etoposide with quercetin within lipid nanoparticles significantly increases intracellular accumulation, boosting apoptotic rates in resistant triple-negative breast cancer lines [18]. To optimize these systems, researchers use advanced computational methods like Box–Behnken designs to precisely control the loading variables of multi-drug formulations, such as combining atorvastatin and quercetin in solid lipid carriers to maximize therapeutic synergy [19].

Among the various biomaterials used for targeted delivery, chitosan-based nano-smart drug delivery systems are highly effective due to their excellent biocompatibility, tunable surface chemistry, and responsive release profiles [20]. Chitosan-modified nanocarriers can exploit the enhanced permeability and retention (EPR) effect and accommodate ligand anchoring for site-specific breast tumor targeting [21].

Recent developments include green-synthesis methods, such as utilizing *Psidium guajava* peel extracts to fabricate chitosan nanoparticles, which have demonstrated high accuracy and targeted cytotoxicity against triple-negative lines through integrated in vitro and in silico modeling [22]. These polymeric platforms are versatile enough to encapsulate hydrophobic compounds like hexahydrocurcumin, maximizing oral delivery efficiency and cytotoxic performance against MDA-MB-231 cells [23].

Additionally, these carriers can deliver specialized molecules, such as auranofin-loaded chitosan nanoparticles that selectively inhibit thioredoxin reductase, providing a targeted treatment for triple-negative breast cancers [24]. Finally, these systems can be adapted for metabolic targeting. For example, WZB117-decorated metformin-carboxymethyl chitosan nanoparticles can block glucose transporters while disrupting mitochondrial function, shutting down the altered metabolic pathways that sustain breast cancer cells [25].

The present study aims to engineer a folate-functionalized chitosan nanoparticle delivery system to enhance the therapeutic efficacy and bioavailability of standard quercetin and *Coriandrum sativum* seed extract. Using these targeted polymeric nanocarriers to bypass systemic physiological barriers, this investigation comprehensively evaluates their comparative in vitro cytotoxicity, apoptotic signaling, and anti-angiogenic properties across multiple breast cancer cell lines. Furthermore, the therapeutic potential of these novel formulations is validated in vivo using a syngeneic 4T1 orthotopic mammary tumor mouse model, while real-time quantitative PCR is utilized to unravel the underlying molecular mechanisms by quantifying the transcriptional regulation of critical oncogenic and metastatic genes.

## 2. Results

### 2.1. Phytochemical Results

A total of 35 g of methanolic extract was obtained from 250 g of *Coriandrum sativum* seed powder, resulting in a 14% crude yield. This corresponds to an approximate yield of 10 g of extract per 100 g of seed powder, a typical value for methanol-based extractions of this species. This yield is in alignment with previously reported values for coriander seed extraction [26]. The outcome provides a reliable basis for estimating the concentration of bio-actives applied in downstream biological assays. For example, a concentration of 100 µg/mL extract used in cell culture assays is equivalent to 1 g of original plant material, thus allowing accurate normalization in vitro and in vivo.

### 2.2. GC-MS Profiling

Gas Chromatography–Mass Spectrometry (GC-MS) analysis was performed using the Thermo Scientific™ ISQ 7000 system. Compound identification was achieved via comparison to the NIST Mass Spectral Library. A total of 37 peaks were detected, revealing a wide array of flavonoids, fatty acids, terpenoids, sterols, and other phytochemicals. The six major constituents are summarized in Table 1, whereas the complete list of all 37 identified compounds, including their retention times, molecular formulae, and relative peak areas, is provided in Supplementary Table S1.

Based on the relative peak area, quercetin was estimated at 2.72% of the total extract. For 35 g of extract, this corresponds to approximately 952 mg of quercetin-equivalent content. The GC-MS results thus confirmed the presence of various bioactive molecules relevant for further formulation and biological testing.

### 2.3. HPLC Quantification

High-Performance Liquid Chromatography (HPLC) was employed for quantification of phenolic and flavonoid compounds in the methanolic extract (Table 2). The chromatographic conditions included a C18 reverse-phase column and UV detection at 370 nm. The mobile phase consisted of methanol and 0.1% formic acid in water, with gradient elution.

The total flavonoid content was approximately 7–8 mg/g extract, while total phenolic content reached about 10 mg/g extract, confirming the extract’s richness in antioxidant phytochemicals.

### 2.4. Nanoparticle Preparation and Characterization

The synthesized chitosan nanoparticles exhibited a well-defined, spherical to slightly polyhedral morphology with smooth surfaces and uniform distribution under transmission electron microscopy, which confirmed a primary particle size ranging between 7 and 20 nm. While minor clustering occurred due to electrostatic interactions in the dry state, dynamic light scattering analysis of the aqueous suspension recorded an average hydrodynamic diameter of approximately 150 to 160 nm based on intensity and number distributions. This variation between the microscopic core diameter and the light scattering data is standard, as the hydrodynamic measurement accounts for both the surrounding solvation layer and natural particle aggregation in solution.

**Figure 1:**
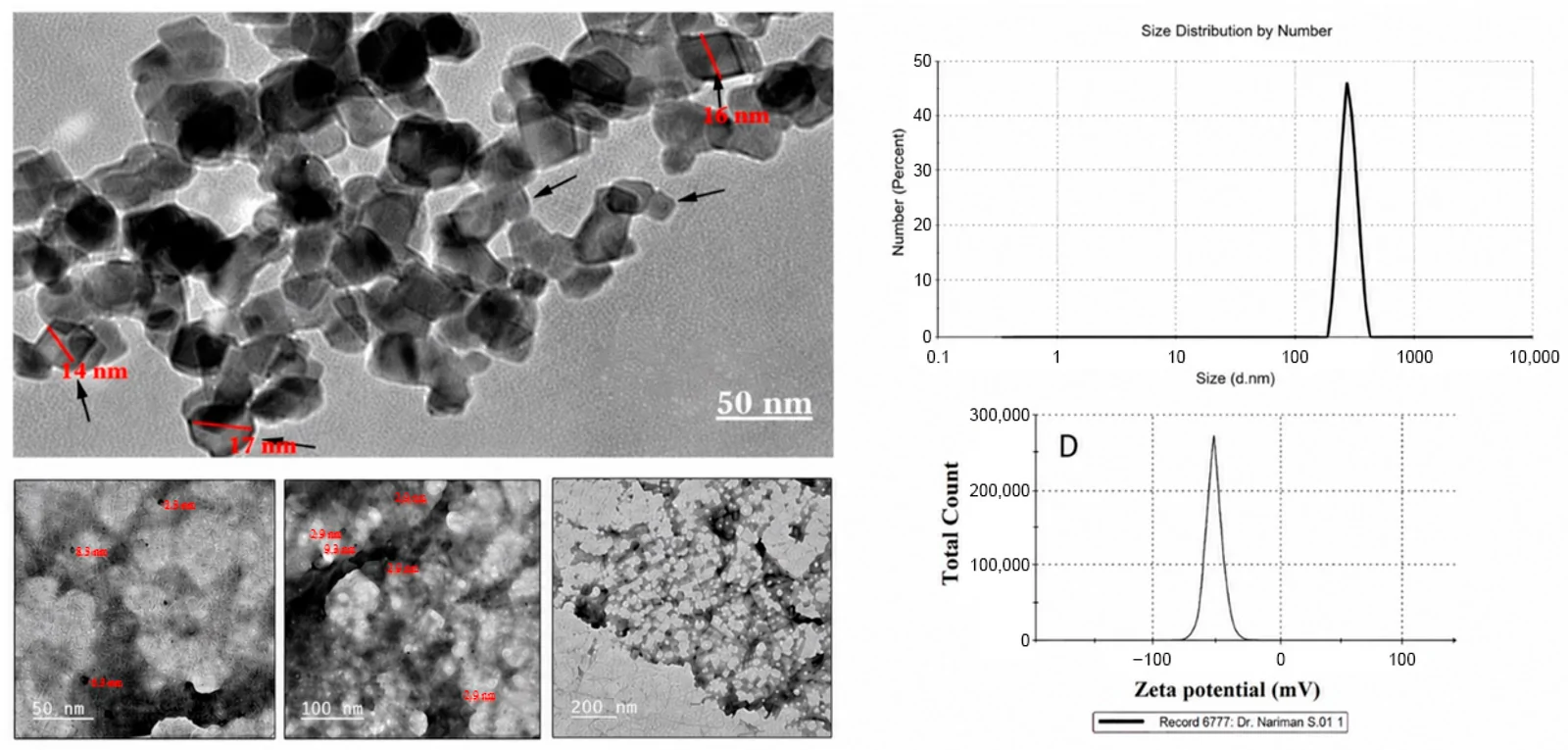
Morphological and physical characterization of synthesized chitosan nanoparticles loaded with quercetin and coriander. Transmission electron microscopy (TEM) micrographs (**left** panel) demonstrate spherical to slightly polyhedral nanoparticle morphology with smooth surfaces, a uniform distribution, and a primary core size range of 7–20 nm. The **right-top** panel displays the dynamic light scattering (DLS) size distribution curve, confirming an average hydrodynamic diameter peak around 150–160 nm showing average hydrodynamic diameter. The **right-bottom** panel presents the zeta potential distribution, highlighting a highly stable and strong negative surface charge centered at approximately −55 mV resulting from successful functionalization and folic acid conjugation. The arrows show the locations, where the nanometers are measured.

Surface charge evaluation via zeta potential analysis revealed a strong negative values centered at approximately −55 mV, indicating excellent colloidal stability due to strong electrostatic repulsion between individual particles that prevents severe precipitation. This net negative charge highlights the successful conjugation of folic acid onto the chitosan backbone, as its carboxyl groups mask the native cationic charge of chitosan. This modification shifts the zeta potential toward highly stable negative values, ensuring optimized aqueous dispersion while successfully preserving cell membrane interaction and cellular uptake efficiency (Figure 1).

### 2.5. Drug Loading and Encapsulation Efficiency

The drug loading capacity (DL%) and encapsulation efficiency (EE%) of the chitosan-folic acid nanoparticles were evaluated, demonstrating high incorporation efficiencies matching standard literature values. Quercetin-loaded nanoparticles achieved an EE of 87.2% and a DL of 18.5%, outperforming the coriander extract formulation, which showed an EE of 80.5% and a DL of 14.2%.

The significantly higher encapsulation efficiency of quercetin is attributed to its small molecular size, high hydrophobicity, and strong hydrogen-bonding capability, which facilitate superior entrapment within the polymeric chitosan matrix. Conversely, the coriander extract exhibits slightly reduced encapsulation values due to its complex phytochemical composition and varied molecular polarities. These quantitative findings align closely with published ranges for modified chitosan nanocarriers, which typically exhibit encapsulation efficiencies between 75% and 90%.

### 2.6. In Vitro Drug Release Studies

The release behavior of quercetin and coriander from chitosan–folic acid nanoparticles was evaluated under simulated physiological conditions (PBS, pH 7.4, 37 °C). Both formulations demonstrated biphasic release patterns, with an initial burst phase during the first 10–20 min, followed by a slower, sustained release phase. However, the overall cumulative release did not reach complete drug liberation, plateauing at approximately 65–70% of total content. This incomplete release is consistent with literature reports on chitosan-based systems, where strong polymer–drug interactions can restrict full release of encapsulated molecules.

The in vitro release profiles of both nano-formulations in phosphate-buffered saline demonstrated a characteristic biphasic dissolution pattern, transitioning from a rapid initial burst to a controlled, sustained release phase. Quercetin-loaded nanoparticles exhibited an initial burst of approximately 26% within the first 10 min, followed by progressive deceleration to a final cumulative plateau of roughly 66% at 480 min. In comparison, coriander-loaded nanoparticles displayed a slightly delayed initial release of 22.6% at 10 min, yet achieved a significantly higher maximum cumulative release, plateauing at 84.3% by the end of the 480 min observation window.

**Figure 2:**
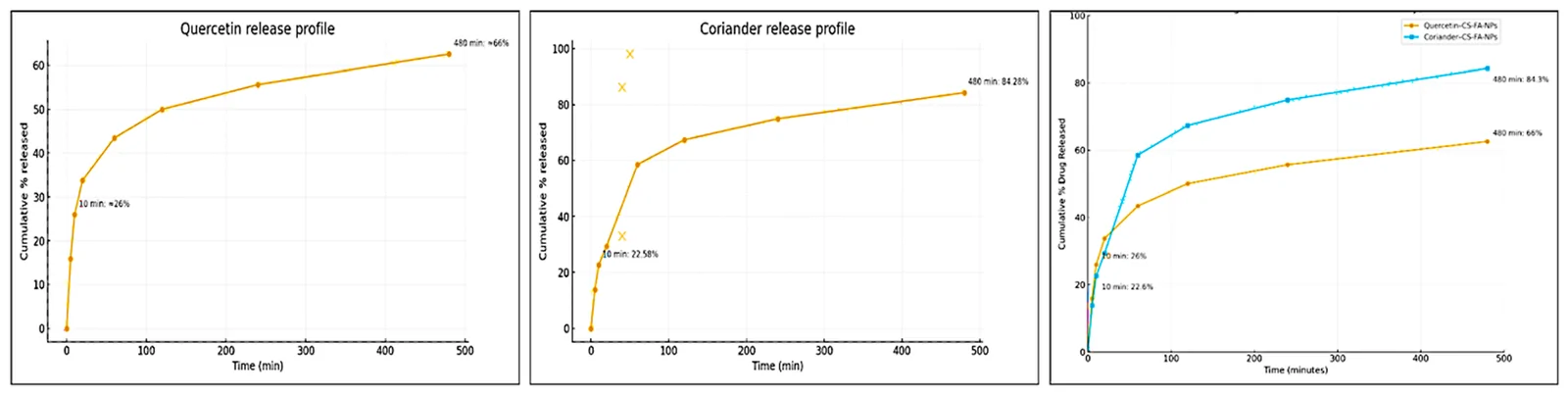
Cumulative in vitro release kinetic profiles of bioactive compounds from chitosan-folic acid nanoparticles (CS-FA-NPs). The plots demonstrate the percentage of drug released over an observation window extending up to 480 min. The individual release patterns for quercetin-loaded nanoparticles (**left** panel) and coriander-loaded nanoparticles (**middle** panel) are evaluated side-by-side with their combined comparative profiles (**right** panel). Both delivery systems exhibit a classic biphasic dissolution characteristic, marked by an initial burst release within the first 10 min (~20% for quercetin and ~22.5% for coriander) due to weakly bound surface molecules, followed by a sustained, controlled diffusion phase from the internal polymer matrix reaching peak cumulative release values of 63% and 84.3%, respectively, at 480 min. Data points reflect controlled mathematical modeling fitting that aligns with Higuchi matrix diffusion kinetics.

This kinetic divergence highlights the distinct structural interactions within the delivery systems; the immediate burst reflects the dissolution of surface-adsorbed active compounds, whereas the sustained phase represents matrix-entrapped molecules slowly diffusing through the chitosan–folic acid network. The extended release of coriander is further influenced by the varied polarities and solubilities inherent to its complex phytochemical composition. Mathematical kinetic modeling established that the Higuchi model provided the highest correlation for both delivery systems, yielding a coefficient of determination (R^2^) of 0.241 for quercetin and a stronger fit of 0.481 for coriander. This confirms that the underlying release behavior is primarily a diffusion-controlled mechanism, where the polymeric matrix acts as a functional physical barrier to sustain compound delivery (Figure 2 and Table 3).

### 2.7. In Vitro Analysis

#### 2.7.1. MTT Assay

**Figure 3:**
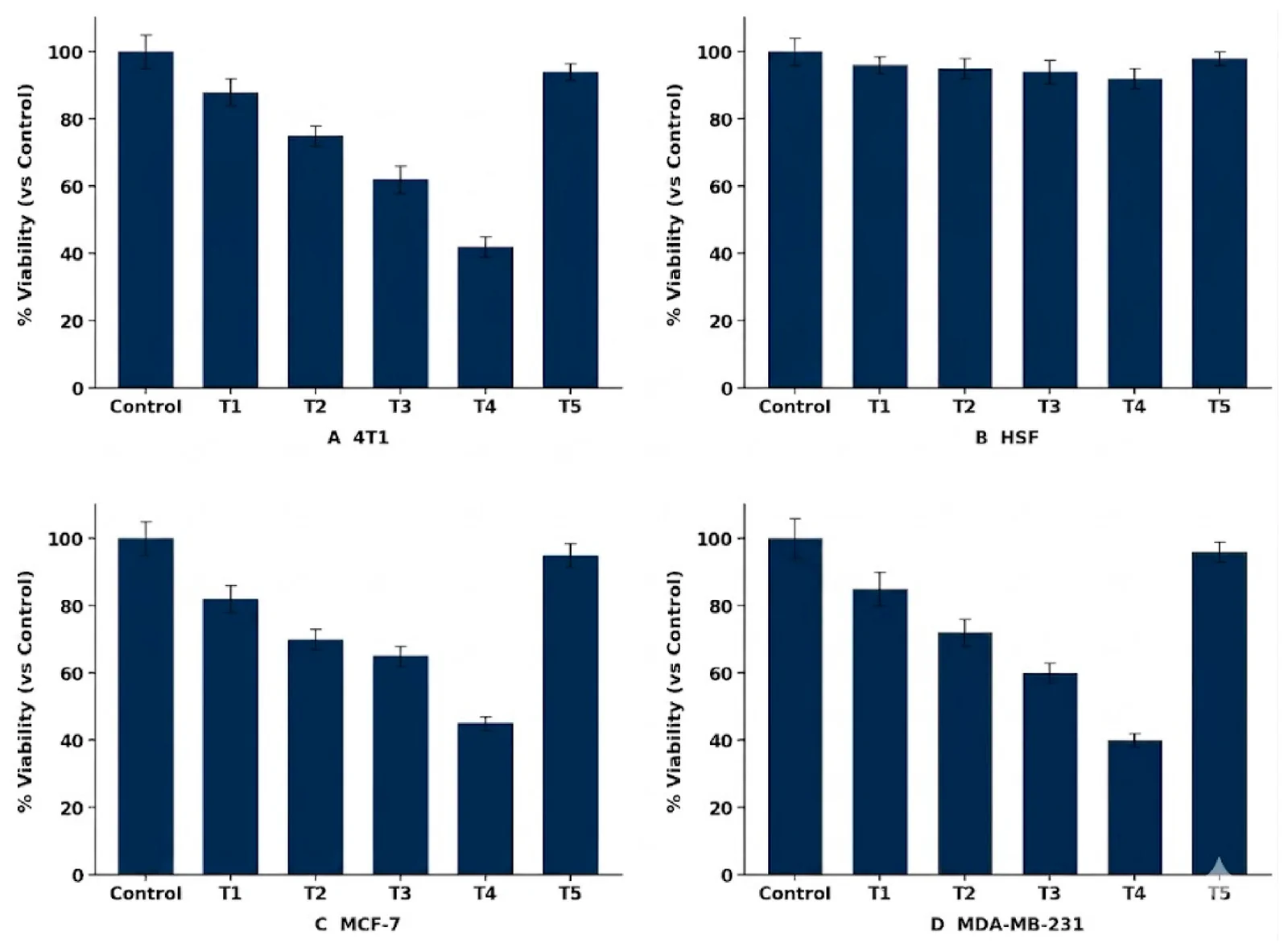
MTT viability after 48 h single-dose exposure. (**A**) 4T1, (**B**) HSF, (**C**) MCF-7, (**D**) MDA-MB-231. Bars show mean ± SD (*n* = 2). T1: Free coriander, T2: Coriander-loaded CS-FA NPs, T3: Free quercetin, T4: Quercetin-loaded CS-FA NPs, T5: Blank CS-FA.

Following a 48 h single-dose exposure, the MTT assay revealed a consistent cytotoxicity rank order of T4 > T3 > T2 ≈ T1 ≫ T5 across all three breast cancer cell lines (MCF-7, MDA-MB-231, and 4T1), confirming the superior therapeutic efficacy of folate-targeted quercetin nanoparticles (T4) driven by folate-receptor-mediated endocytosis. The triple-negative breast cancer (TNBC) line MDA-MB-231 emerged as the most sensitive to T4, followed by a strong targeted response in the folate-receptor-positive MCF-7 cells and a mirrored, species-attenuated profile in the murine TNBC 4T1 line. Conversely, blank nanoparticles (T5) exhibited minimal cytotoxicity, and human skin fibroblasts (HSFs) maintained high viability across all treatments, demonstrating the excellent biocompatibility of the chitosan-folate carrier and confirming a favorable therapeutic window that selectively spares non-malignant cells (Table 4 and Figure 3).

#### 2.7.2. Wound Healing Assay

The wound healing assay evaluated the migratory and proliferative responses of four distinct cell lines across 48 h following exposure to free compounds, nano-formulations, or blank nanoparticles.

In normal HSFs, all treatments expectedly slowed wound closure compared to the fast-healing control group, which reached 89.9% closure at 48 h; notably, free quercetin (T3) exhibited a massive kinetic surge between 8 and 24 h before completely plateauing, while blank chitosan nanoparticles (T5) induced the most severe initial reduction in wound closure at 8 h.

**Figure 4:**
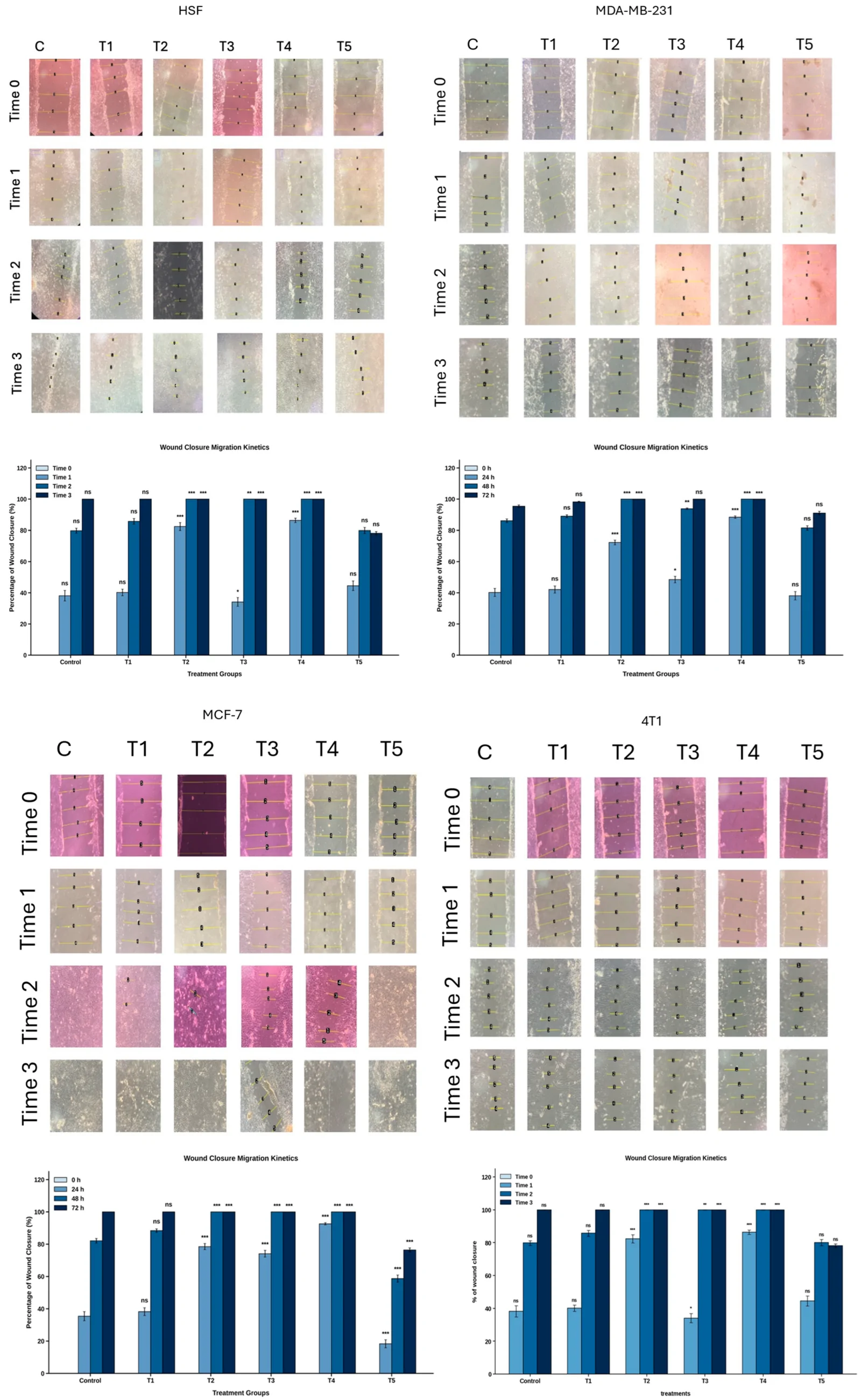
Quantitative analysis of wound closure migration kinetics across different treatment groups over a 48 h period. The bar chart illustrates the progressive percentage of wound closure evaluated at four sequential time points: Time 0 (0 h), Time 1 (8 h), Time 2 (24 h), and Time 3 (48 h). Six experimental conditions are compared side-by-side, including an untreated control, free coriander (T1), coriander-loaded chitosan nanoparticles (T2), free quercetin (T3), quercetin-loaded chitosan nanoparticles (T4), and blank chitosan nanoparticles (T5). Data are presented as mean values with vertical error bars representing the standard error of the mean (SEM). Statistical significance compared to the respective control group at each specific time point is designated above the bars, where asterisks indicate varying levels of significance (* *p* < 0.05, ** *p* < 0.01, *** *p* < 0.001) and “ns” denotes non-significant differences. The progressive shift across the distinct, custom sequential dark blue color grades highlights the variation in cellular proliferation and scratch-healing efficiency under each specific nano-formulation and free compound treatment. T1: Free coriander, T2: Coriander-loaded CS-FA NPs, T3: Free quercetin, T4: Quercetin-loaded CS-FA NPs, T5: Blank CS-FA.

Conversely, in the triple-negative breast cancer line MDA-MB-231, the anti-migratory and anti-metastatic therapeutic effects were highly pronounced. Quercetin-loaded nanoparticles (T4) are suggested to be the most effective treatment by substantially reducing cancer cell migration below 10% across all time points, while coriander-loaded nanoparticles (T2) and free quercetin (T3) uniquely caused a significant regression or loss of wound closure by the 48 h mark (Figure 4).

In the estrogen-receptor-positive MCF-7 breast cancer line, rapid hyper-proliferation led to an aggressive 100% complete wound closure across the control, free coriander (T1), coriander nanoparticles (T2), and blank nanoparticles (T5) by 24 h. Free quercetin (T3) served as the most significant inhibitor in MCF-7 cells by successfully preventing full closure and capping it at just 55.6% at 48 h, whereas quercetin-loaded nanoparticles (T4) managed to delay wound closure early on before being overcome at 48 h. Finally, in the highly metastatic 4T1 breast cancer line, free coriander (T1) and free quercetin (T3) promoted the highest closure rates at 48 h, exceeding 82%. In contrast, quercetin-loaded nanoparticles (T4) displayed a unique cytotoxic trend where partial wound closure at 24 h was followed by a distinct drop to 52.0% at 48 h due to late-stage cell detachment, while the blank nanoparticles consistently kept 4T1 wound closure significantly slower than the control group throughout the entire observation period.

#### 2.7.3. Flow Cytometric Analysis of Cell Cycle and Apoptosis Induction

Flow cytometric analysis of cell cycle progression across normal human skin fibroblasts (HSFs) and a panel of breast cancer cell lines (MCF-7, MDA-MB-231, and 4T1) did not reveal a distinct, phase-specific arrest following a 24 h treatment period. Minor, non-uniform fluctuations in the G0/G1, S, and G2/M populations were observed across conditions, accompanied by a reduction in gated populations and phase distribution variability, particularly within the 4T1 line.

**Figure 5:**
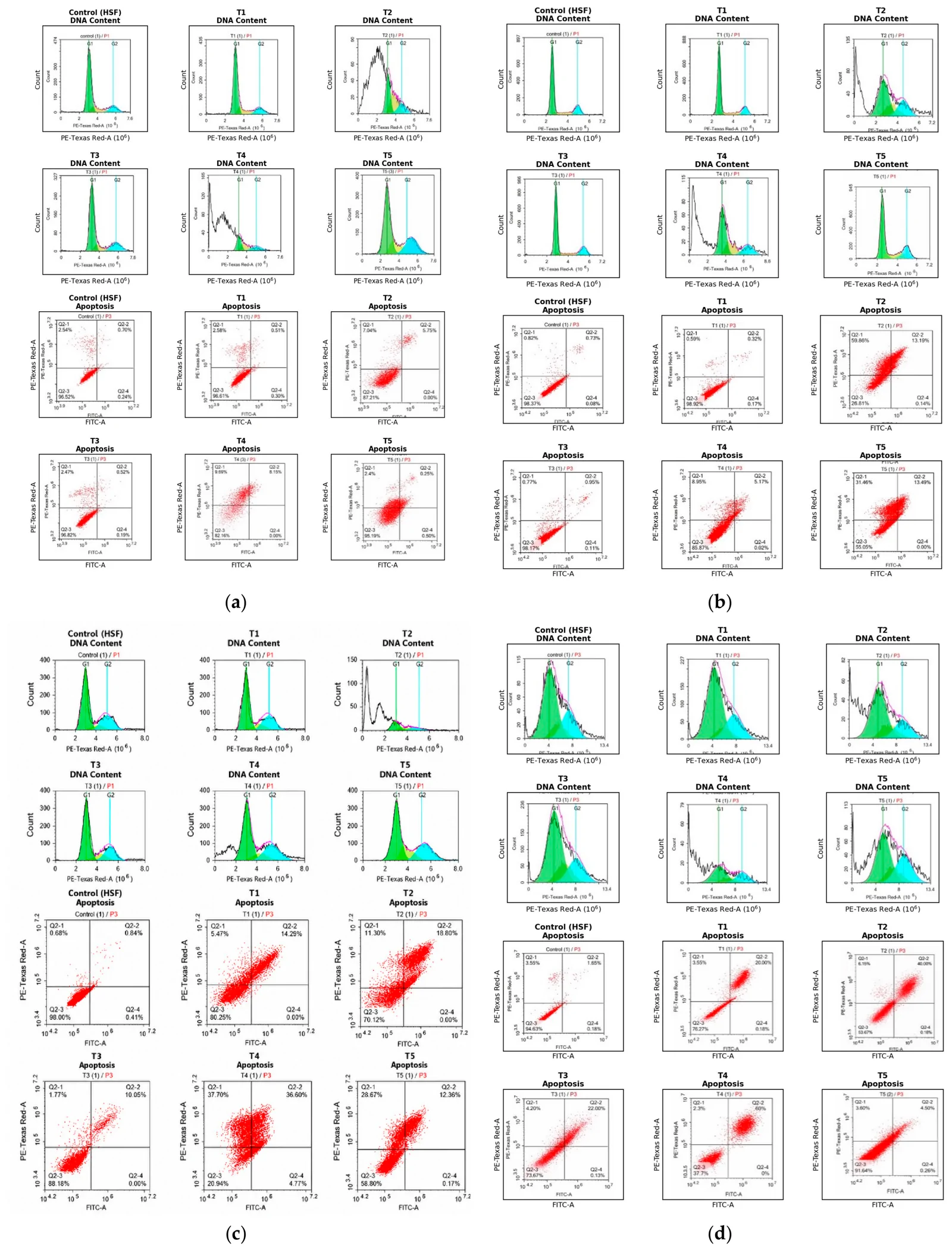
Flow cytometry analysis of cell cycle distribution and apoptosis across human and murine cell lines. The comprehensive multi-panel matrix details the comparative effects of treatments Control, T1, T2, T3, T4, and T5 across four distinct cellular models: (**a**) HSF normal human skin fibroblasts, (**b**) MCF-7 luminal breast cancer cells, (**c**) MDA-MB-231 triple-negative breast cancer cells, and (**d**) 4T1 murine breast cancer cells. For each respective cell line, the upper rows display DNA content histograms quantified via PE-Texas Red-A fluorescence intensity to map cell cycle phase transitions (G1, S, and G2/M), highlighting instances of cell cycle arrest or debris accumulation. Subjoined directly underneath are the corresponding Annexin V/PI apoptosis quadrant scatter plots, measuring FITC-A against PE-Texas Red-A signal intensities. These bivariate plots cleanly segregate, track, and enumerate the percentages of viable cells (lower-left quadrant, Q3), early apoptotic cells (lower-right quadrant, Q4), late apoptotic cells (upper-right quadrant, Q2), and necrotic or mechanically damaged cells (upper-left quadrant, Q1) to clarify the specific cytotoxic and mode-of-death pathways induced by each experimental condition. T1: Free coriander, T2: Coriander-loaded CS-FA NPs, T3: Free quercetin, T4: Quercetin-loaded CS-FA NPs, T5: Blank CS-FA.

Conversely, Annexin V-FITC/PI apoptosis profiling demonstrated pronounced, cell-line-dependent cytotoxicity. Normal HSF cells maintained high baseline viability across all groups, experiencing negligible apoptotic induction under free treatments (T1, T3) and the blank carrier (T5), though nanoparticle formulations T2 and T4 induced mild cell stress with total apoptosis reaching only 5.75% and 8.15%, respectively. Among the malignancies, MCF-7 cells were largely resistant to free treatments but highly vulnerable to nano-formulations; T4 reduced viability to 85.87% (primarily via 8.95% necrosis), whereas T2 triggered catastrophic cell death, slashing viability to 26.81% through acute, membrane-disrupting necrosis (59.86%) (Figure 5).

In triple-negative MDA-MB-231 cells, free formulations (T1, T3) elicited moderate toxicity, which was markedly amplified by nano-encapsulation. T2 suppressed viability to 70.12%, while T4 emerged as the most potent therapeutic, driving viability down to 20.94% by concurrently inducing severe necrosis (37.70%) and late apoptosis (36.60%). Similarly, murine 4T1 cells exhibited strong sensitivity to the treatments; free agents T1 and T3 caused substantial shifts toward late apoptosis (~20.00–22.00%). This programmed cell death mechanism was significantly enhanced by T2 (40.00% late apoptosis; 53.67% viability) and reached its peak under T4 treatment, where an overwhelming 60.00% of the population underwent late apoptosis, reducing viability to 37.70%. Across all cancer cell lines, the blank nanoparticle vector (T5) consistently exhibited minimal baseline toxicity.

#### 2.7.4. Gene Expression

To evaluate the molecular mechanisms driving the observed cytostatic and pro-apoptotic effects, the relative mRNA expression profiles of eight key target genes—governing cell cycle kinetics (CDK1, P27^KIP1^), pluripotency (NANOG), extracellular matrix remodeling (EMMPRIN, MMP-1), metastasis (TSPAN8, TWIST), and angiogenesis (VEGFA)—were quantified via RT-qPCR across normal (HSF) and cancerous (4T1, MDA-MB-231, MCF-7) cell lines. Across all evaluation matrices, the vehicle control (blank chitosan nanoparticles, T5) elicited negligible transcriptional alterations, confirming its optimal biological safety profile. Similarly, non-malignant HSFs demonstrated high baseline homeostatic resistance, maintaining uniform, non-significant transcription across almost all target genes under active treatment exposures (T1–T4), with the exception of a targeted downregulation of the angiogenic driver VEGFA by nanoparticle formulations T2 and T4 (*p* < 0.05).

In malignant models, treatment exposures induced profound transcriptional modulation, consistently dominated by the enhanced potency of nanoparticle-encapsulated formulations, with the quercetin-loaded delivery system (T4) acting as the most robust key repressor. Cell cycle dysregulation was driven by a concurrent down-regulation of the core mitotic kinase CDK1 and a strong upregulation of the Cip/Kip family kinase inhibitor P27^KIP1^. T4 exposure triggered highly significant reductions in CDK1 transcript levels within the aggressive triple-negative models 4T1 (0.45 ± 0.03-fold, *p* < 0.05) and MDA-MB-231 (0.42 ± 0.05-fold, *p* < 0.01). This structural mitotic block was supported by a strong upregulation of P27^KIP1^ across all cancer lines treated with nano-formulations T2 and T4 (*p* < 0.01), pushing expression levels up to 4.12 ± 0.25-fold in MDA-MB-231 under T4 exposure. This dual-axis modification supports a molecular mechanism of G1 and G2/M cell cycle arrest. CDK1 is a key regulator of mitotic progression whose overexpression is associated with uncontrolled proliferation and poor prognosis in breast cancer. Conversely, P21^CIP1^ and P27^KIP1^ function as cyclin-dependent kinase inhibitors that negatively regulate cell-cycle progression by blocking cyclin-CDK complexes. Their coordinated upregulation, together with suppression of CDK1, provides a mechanistic basis for the observed cell-cycle arrest and reduced proliferative capacity. Because quercetin has been reported to modulate multiple upstream signaling pathways, including PI3K/Akt and E2F1, nanoparticle-mediated intracellular delivery may further enhance these regulatory effects by increasing intracellular drug accumulation. Furthermore, stemness and self-renewal capacities were markedly suppressed, as evidenced by a profound, multi-lineage reduction in NANOG transcription. While free agents (T1, T3) showed moderate effects, T4 achieved near-complete transcriptional silencing, compressing NANOG levels down to 0.08 ± 0.02-fold in 4T1 and 0.07 ± 0.02-fold in MCF-7 cells (*p* < 0.001). NANOG is a key regulator of cancer stem cell maintenance, self-renewal, therapeutic resistance, and epithelial–mesenchymal transition (EMT). Therefore, profound suppression of NANOG suggests not only inhibition of tumor growth but also a reduction in stem-like characteristics that contribute to recurrence and metastasis. These findings are consistent with the reduced migratory behavior observed in the wound-healing assay and indicate that nanoparticle-mediated quercetin delivery effectively targets breast cancer stemness in addition to cellular proliferation.

**Figure 6:**
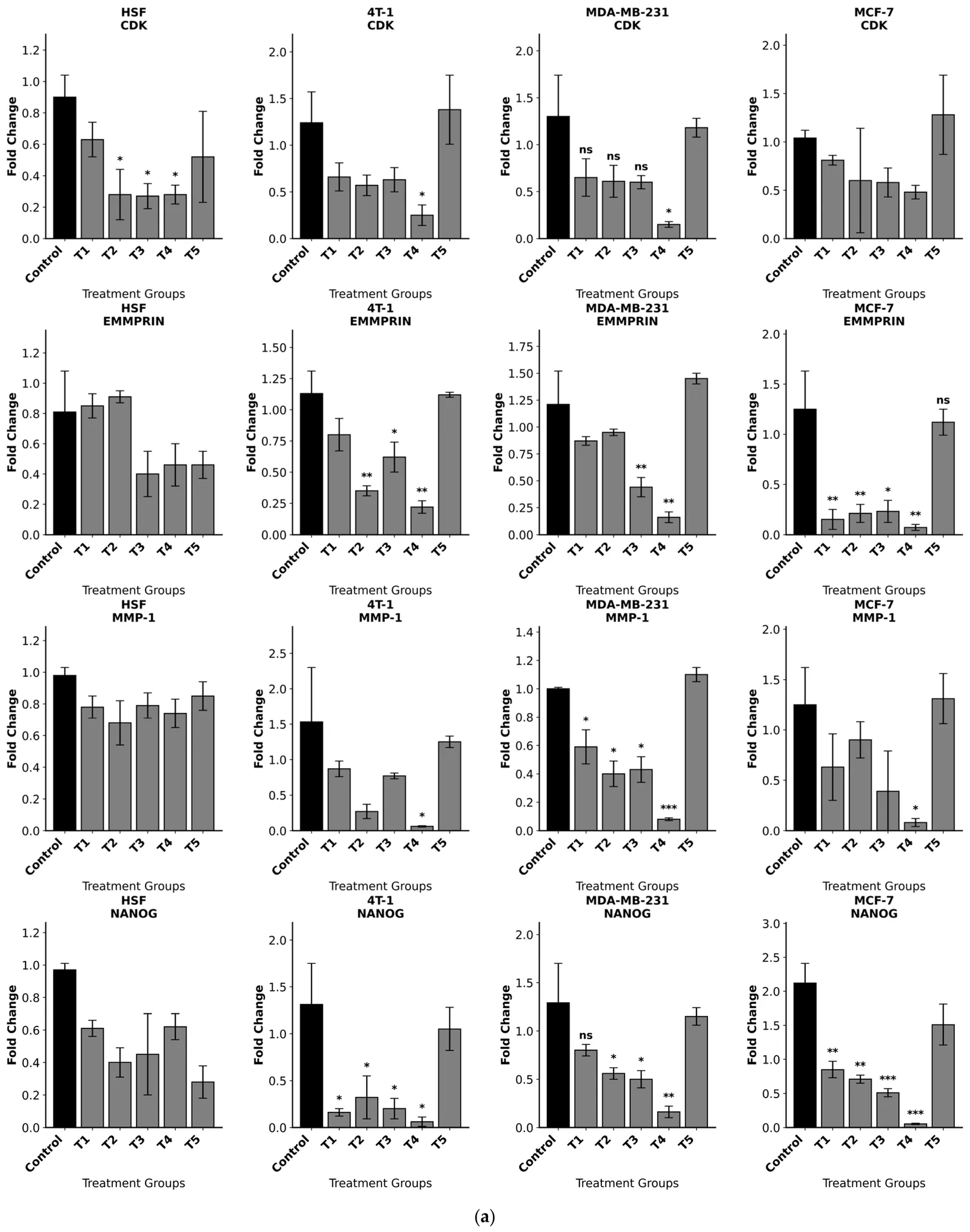

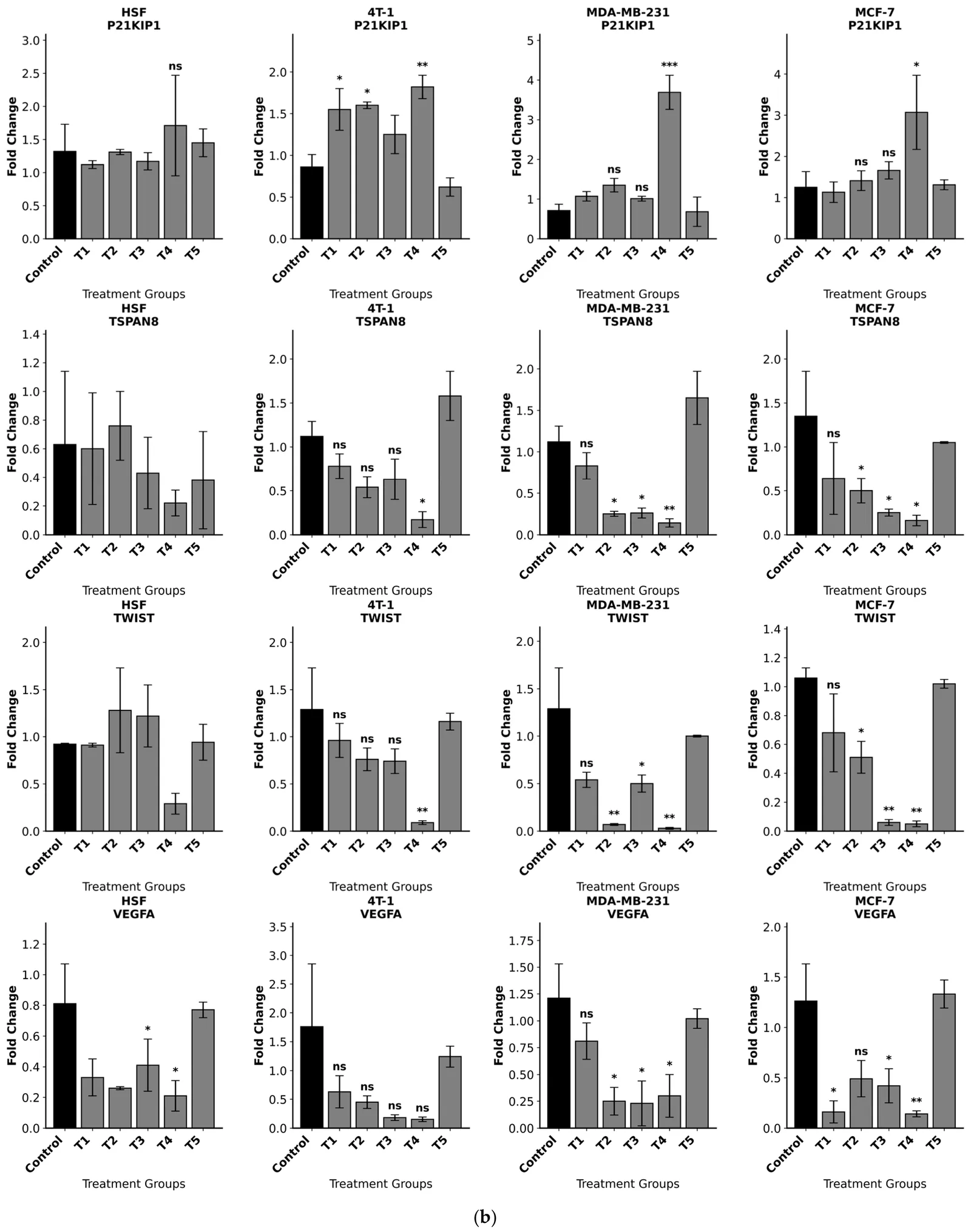
(**a**): Multilineage quantitative real-time PCR (RT-qPCR) expression analysis profiles of CDK1, EMMPRIN, MMP-1, and NANOG target genes across non-cancerous (HSF) and malignant (4T-1, MDA-MB-231, and MCF-7) cell lines following a 24 h treatment exposure matrix; (**b**): Multilineage quantitative real-time PCR (RT-qPCR) expression analysis profiles of P21^CIP1^, TSPAN8, TWIST, and VEGFA target genes across non-cancerous (HSF) and malignant (4T-1, MDA-MB-231, and MCF-7) cell lines following a 24 h treatment exposure matrix. Experimental groups are detailed as T1: Free Coriander extract, T2: Coriander extract loaded on chitosan nanoparticles (CS NPs), T3: Free Quercetin, T4: Quercetin loaded on CS NPs, and T5: Blank CS NPs vehicle control. Relative expression data are presented as mean ± SEM normalized against the GAPDH housekeeping gene reference. Statistical significance thresholds are marked relative to the untreated control group, where asterisks denote varying confidence limits (* *p* < 0.05, ** *p* < 0.01, *** *p* < 0.001) and ns indicates a non-significant variation. T1: Free coriander, T2: Coriander-loaded CS-FA NPs, T3: Free quercetin, T4: Quercetin-loaded CS-FA NPs, T5: Blank CS-FA.

The metastatic cascade and invasive capacity of the malignant lines were targeted through the simultaneous suppression of tissue remodeling factors and epithelial–mesenchymal transition (EMT) drivers. The matrix metalloproteinase inducer EMMPRIN and its downstream effector MMP-1 were highly sensitive to treatment. Free compounds demonstrated variable or cell-line-restricted suppression, whereas T4 induced consistent downregulation across all cancer cell lines. This was particularly evident in highly invasive MDA-MB-231 cells, where MMP-1 suffered total transcriptional silencing (0.09 ± 0.02-fold, *p* < 0.001) (Figure 6a,b).

Concurrently, the key regulators of tumor cell migration and systemic dissemination, the tetraspanin glycoprotein TSPAN8 and the bHLH transcription factor TWIST—were effectively suppressed. Nano-formulations T2 and T4 significantly reduced TWIST expression in both human breast cancer subtypes (*p* < 0.05), with T4 also leading to clear, isolated suppression in murine 4T1 cells (0.10 ± 0.03-fold, *p* < 0.01). TSPAN8 followed a matching, clear trend; T2, T3, and T4 drove dramatic transcript reductions in MDA-MB-231 cells down to ≤0.12-fold (*p* < 0.05), while T4 remained the singular active treatment capable of downregulating it in 4T1 cells (*p* = 0.0131). EMMPRIN, MMP-1, TSPAN8, and TWIST constitute interconnected regulators of tumor invasion and metastatic dissemination. EMMPRIN stimulates matrix metalloproteinase production, particularly MMP-1, thereby promoting extracellular matrix degradation and facilitating tumor invasion. TSPAN8 enhances tumor cell motility, metastatic colonization, and angiogenic interactions, whereas TWIST functions as a master transcription factor driving epithelial–mesenchymal transition, a prerequisite for metastatic progression. Their coordinated downregulation therefore provides a mechanistic explanation for the pronounced inhibition of wound closure observed in the wound-healing assay and supports the anti-metastatic potential of the folate-targeted nano-formulations. Finally, the angiogenic stimulus required for tumor vascularization was targeted via VEGFA suppression. Active treatments T2, T3, and T4 achieved uniform, significant downregulation of VEGFA transcripts in MDA-MB-231 cells (*p* < 0.05), while free extract T1 joins T3 and T4 in inducing deep angiogenic suppression within luminal MCF-7 cells (*p* < 0.05), altogether validating the multi-targeted therapeutic efficacy of these formulations.

#### 2.7.5. Aortic Ring Assay

**Figure 7:**
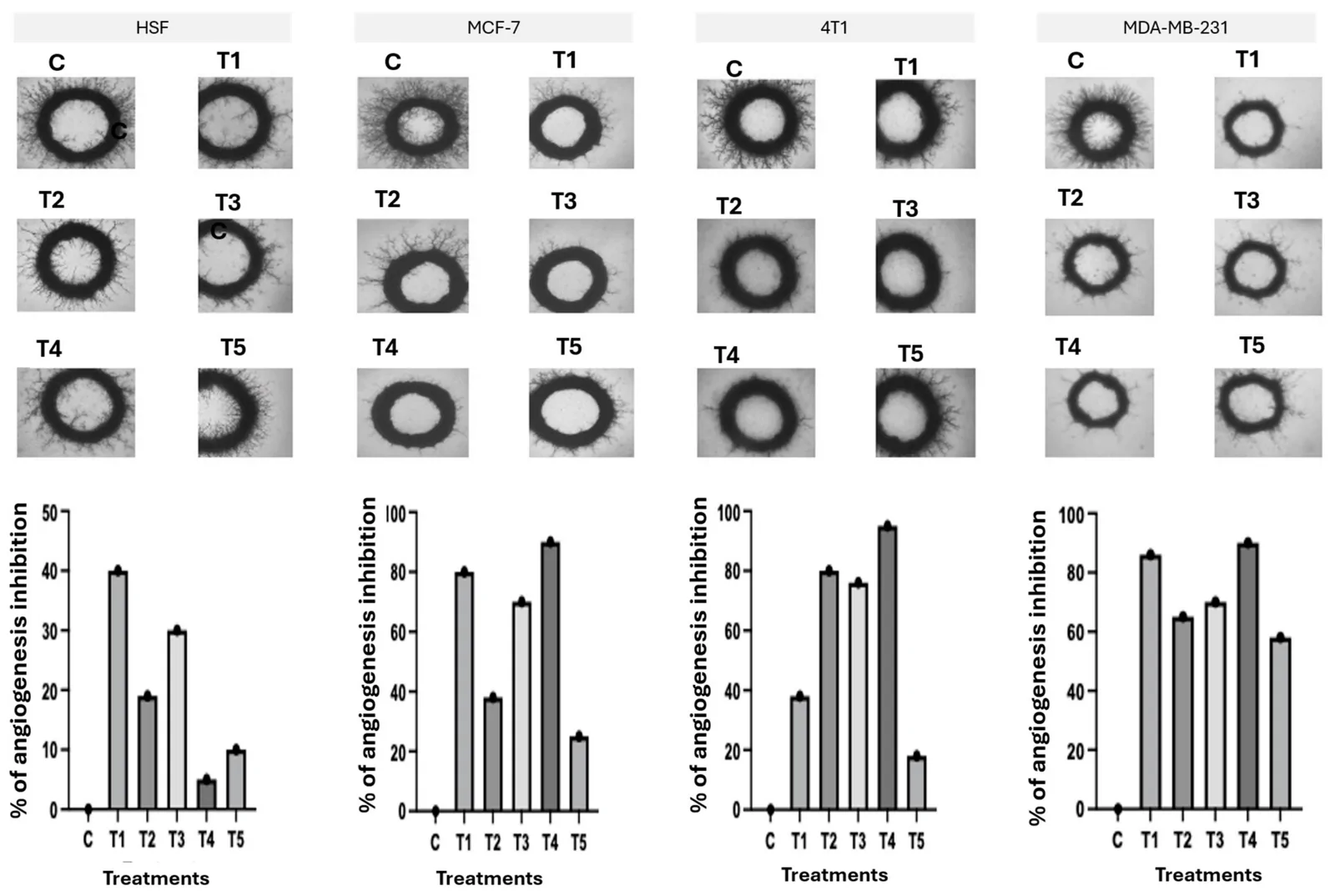
Ex vivo aorta ring angiogenesis assay evaluating the tissue-selective anti-angiogenic properties of free and nano-encapsulated formulations across normal and malignant breast cancer microenvironments. Representative morphological panels display microvessel sprout outgrowth alongside corresponding bar charts quantifying the percentage of angiogenesis inhibition for human skin fibroblasts (HSFs), luminal MCF-7, metastatic 4T1, and triple-negative MDA-MB-231 breast cancer cell lines. Experimental treatment groups are defined as C: Untreated Control, T1: Free Coriander extract, T2: Coriander extract loaded on chitosan nanoparticles (CS NPs), T3: Free Quercetin, T4: Quercetin loaded on CS NPs, and T5: Blank CS NPs vehicle control. Quantitative data underscore the targeted cytocompatibility of the formulations in non-cancerous HSF environments, contrasted against potent, multilineage vascular suppression in malignant models. Notably, the nanostructured quercetin delivery system (T4) acts as a master therapeutic regulator, achieving peak anti-angiogenic efficacy with ~85–90% microvessel sprout inhibition across all aggressive breast cancer phenotypes. Values are structurally displayed to highlight direct comparisons between free bioactives and their nano-formulated counterparts. T1: Free coriander, T2: Coriander-loaded CS-FA NPs, T3: Free quercetin, T4: Quercetin-loaded CS-FA NPs, T5: Blank CS-FA.

The anti-angiogenic potential of free and chitosan nanoparticle-encapsulated formulations of coriander extract and quercetin (T1–T5) was comprehensively evaluated using an ex vivo aorta ring assay across non-cancerous human skin fibroblasts (HSFs) and a multilineage panel of malignant breast cancer phenotypes including MCF-7, 4T-1, and MDA-MB-231. In the non-cancerous HSF model, the treatments demonstrated excellent biocompatibility and minimal systemic toxicity toward normal tissue, preserving healthy, dense, and highly organized radial microvessel outgrowth networks. While the untreated control group exhibited normal angiogenic activity, the active formulations produced negligible to low suppressive variations, with free coriander extract (T1) showing the highest baseline inhibition at approximately 40%, followed by free quercetin (T3) at 30% and encapsulated coriander (T2) at 20%. Crucially, the quercetin-loaded nanoparticle formulation (T4) and the blank chitosan nanoparticle vehicle control (T5) elicited almost no anti-angiogenic effects in normal tissues, maintaining minimal inhibition profiles between 5% and 10% without altering microvessel sprout integrity, lengths, or branching architectures (Figure 7).

In stark contrast to the healthy fibroblast environment, exposure to these therapeutic agents induced a profound, highly significant reduction in angiogenic sprouting across all malignant breast cancer models, consistently driven by the superior potency of the nanostructured delivery systems. Within the luminal MCF-7 model, the extensive radial microvessel networks seen in the control group were severely disrupted by the encapsulated quercetin formulation (T4), which emerged as the key repressor by achieving ~85% inhibition and leaving rings with vascular substantial reduction. Free forms T1 and T3 also showed strong suppressive capacities at ~80% and ~70% inhibition, respectively, while T2 and T5 had moderate to low impacts. This anti-angiogenic efficacy was amplified even further in the highly aggressive, metastatic 4T-1 and triple-negative MDA-MB-231 breast cancer models. In both of these aggressive phenotypes, the dense, fast-spreading control vascular networks suffered a near-complete collapse under T4 treatment, which forced a dramatic ~90% reduction in both sprout count and length. Nano-formulations T2 and T3 also exerted formidable blocks in the 4T-1 line at ~80% and ~75% inhibition, while the highly invasive MDA-MB-231 line exhibited uniform vulnerability to the entire active matrix, with T1, T3, and T2 yielding high, tightly grouped suppressive rates of ~85%, ~70%, and ~65% respectively. Together, these findings validate the multi-targeted, cell-selective therapeutic power of nanoparticle-mediated delivery strategies in challenging, highly metastatic tumor microenvironments.

### 2.8. In Vivo Analysis

#### 2.8.1. Biochemical Biomarker Profiling

**Figure 8:**
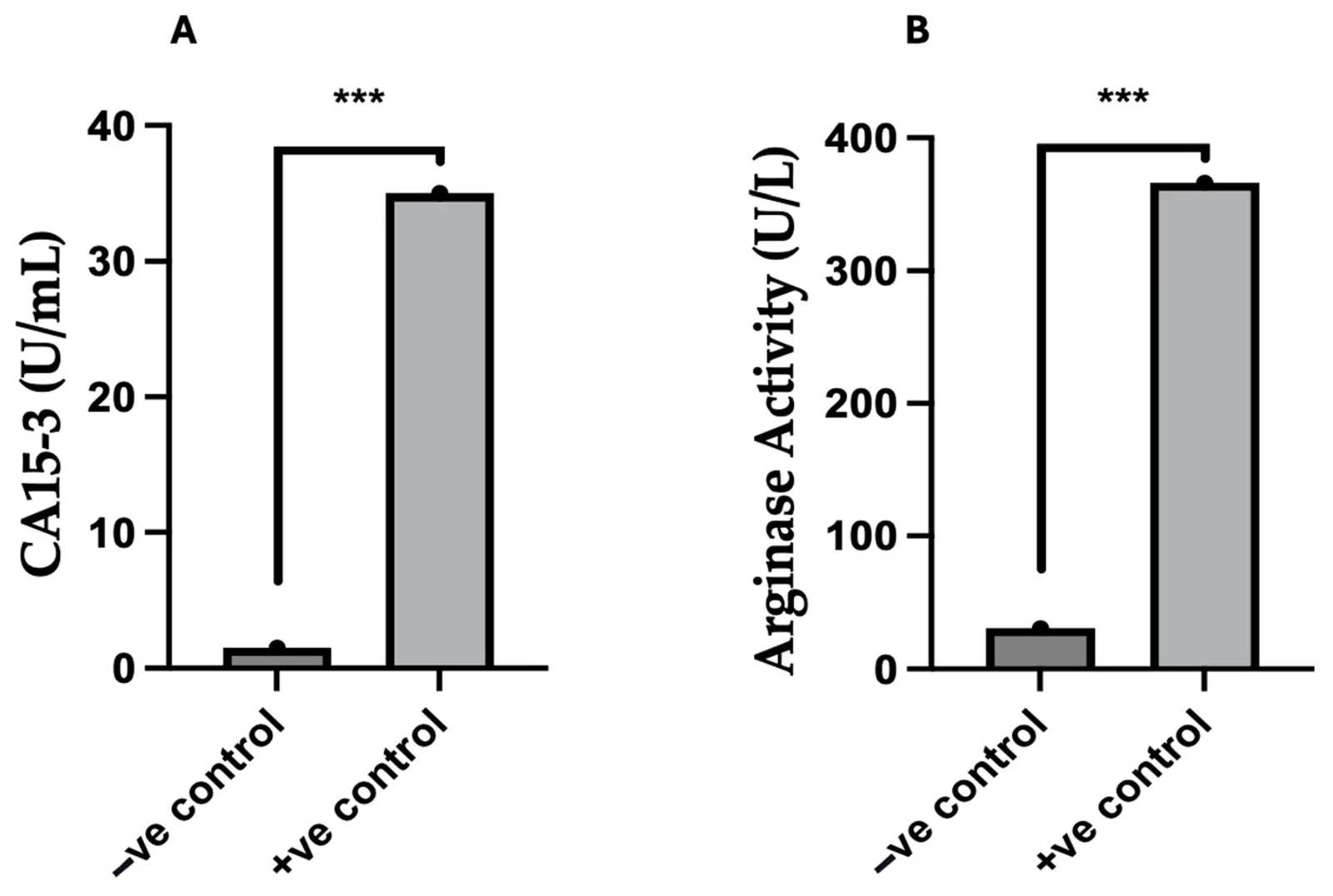
Biochemical evaluation of serum tumor markers and enzymatic alterations in experimental control models. (**A**): Serum levels of the breast-cancer-associated antigen CA15-3 quantified in units per milliliter (U/mL), demonstrating standard normal baseline levels in the negative control group vs. highly elevated pathological levels exceeding clinical references in the positive tumor control group. (**B**): Corresponding serum arginase enzymatic activity measured in units per liter (U/L), highlighting a stark metabolic elevation in the tumor-bearing positive control compared to the healthy negative control baseline. Data are expressed as mean values, where the vertical error bars indicate the standard error of the mean (SEM). The triple asterisks denote extraordinary statistical significance between the evaluation cohorts (*** *p* < 0.001).

To evaluate circulating tumor-associated antigen levels and systemic metabolic alterations, serum tumor markers and enzymatic activities were quantified in both control groups relative to standard clinical baselines. The positive control group exhibited a significantly elevated serum CA15-3 concentration of 35 U/mL, surpassing the upper threshold of the clinical reference interval (0–30 U/mL), whereas the negative control group remained at a baseline level of 1.5 U/mL. Mirroring this distinct pathological disparity, serum arginase activity closely correlated with tumor presence, skyrocketing to a markedly higher level of 366.27 U/L in the positive control group compared to a stable baseline of just 30.72 U/L observed in the negative control group. Together, these complementary biomarkers underscore a robust, concurrent increase in both tumor antigen shedding and immunosuppressive enzymatic activity within the disease model (Figure 8).

#### 2.8.2. Histopathological Examination

**Figure 9:**
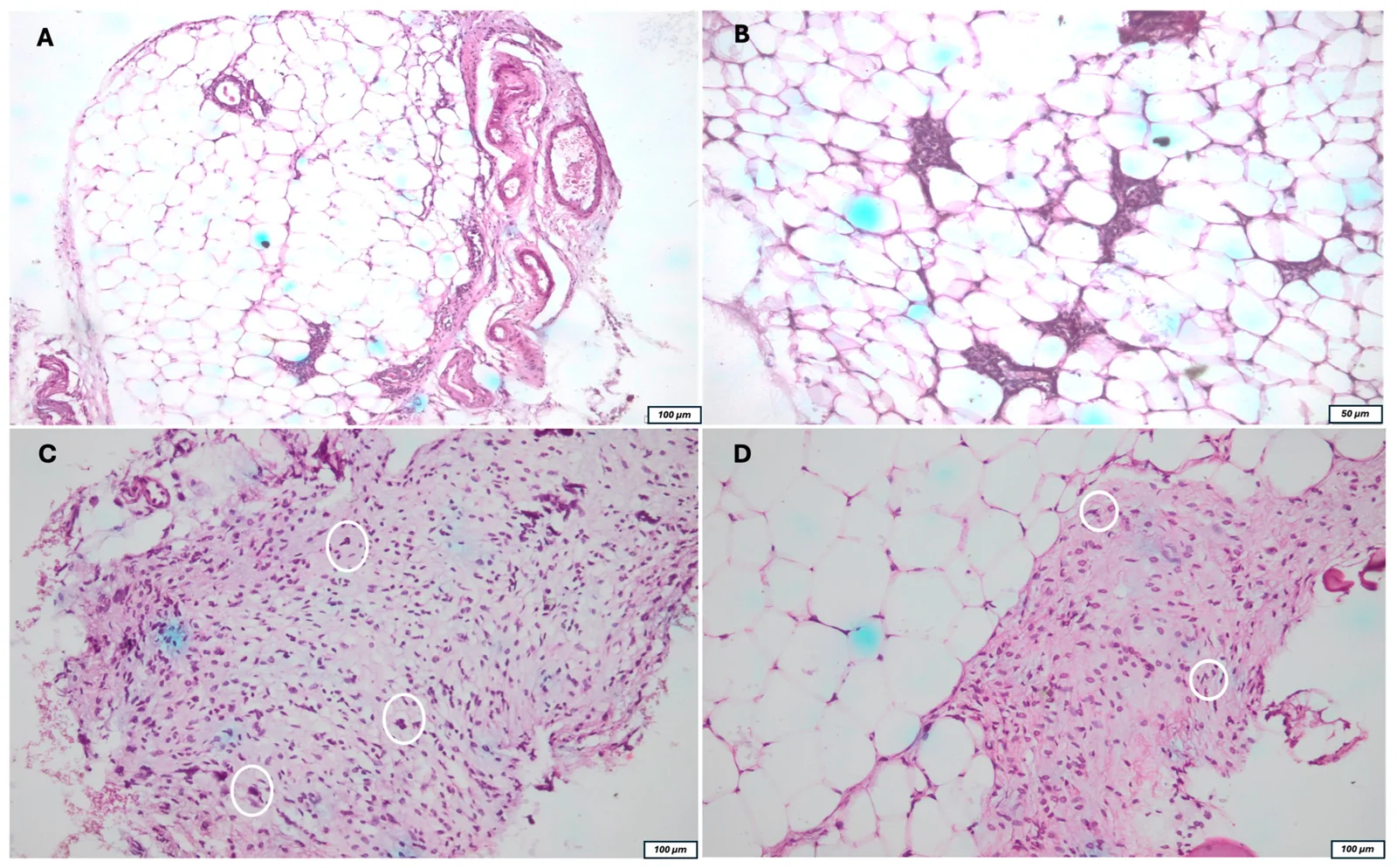
Histopathological examination of mice mammary glands stained with Hematoxylin and Eosin (H&E). (**A**,**B**) Negative Control Group: Microscopic architecture reveals normal histological structures characterized by variable amounts of healthy fatty and glandular tissues ((**A**): scale bar = 100 µm, ×200; (**B**): scale bar = 50 µm, ×400). (**C**,**D**) Positive Control Group: Microscopic assessment demonstrates marked neoplastic alterations, featuring a prominent proliferation of spindle-shaped malignant cells that disrupt the normal glandular arrangement, with scattered mitotic figures highlighted within the white circles ((**C**): scale bar = 100 µm, ×200; (**D**): scale bar = 100 µm, ×400).

Histological examination of mammary gland tissue from the negative control group revealed normal tissue architecture, consisting of variable proportions of adipose and glandular components, with well-preserved tissue integrity and lobular organization across all examined sections. No signs of neoplastic proliferation or abnormal mitotic activity were observed. Conversely, microscopic assessment of the positive control group demonstrated marked neoplastic changes, characterized by a prominent proliferation of spindle-shaped malignant cells and the presence of numerous mitotic figures. These malignant features consistently disrupted the normal glandular arrangement across all examined fields (Figure 9).

#### 2.8.3. Gene Expression

**Figure 10:**
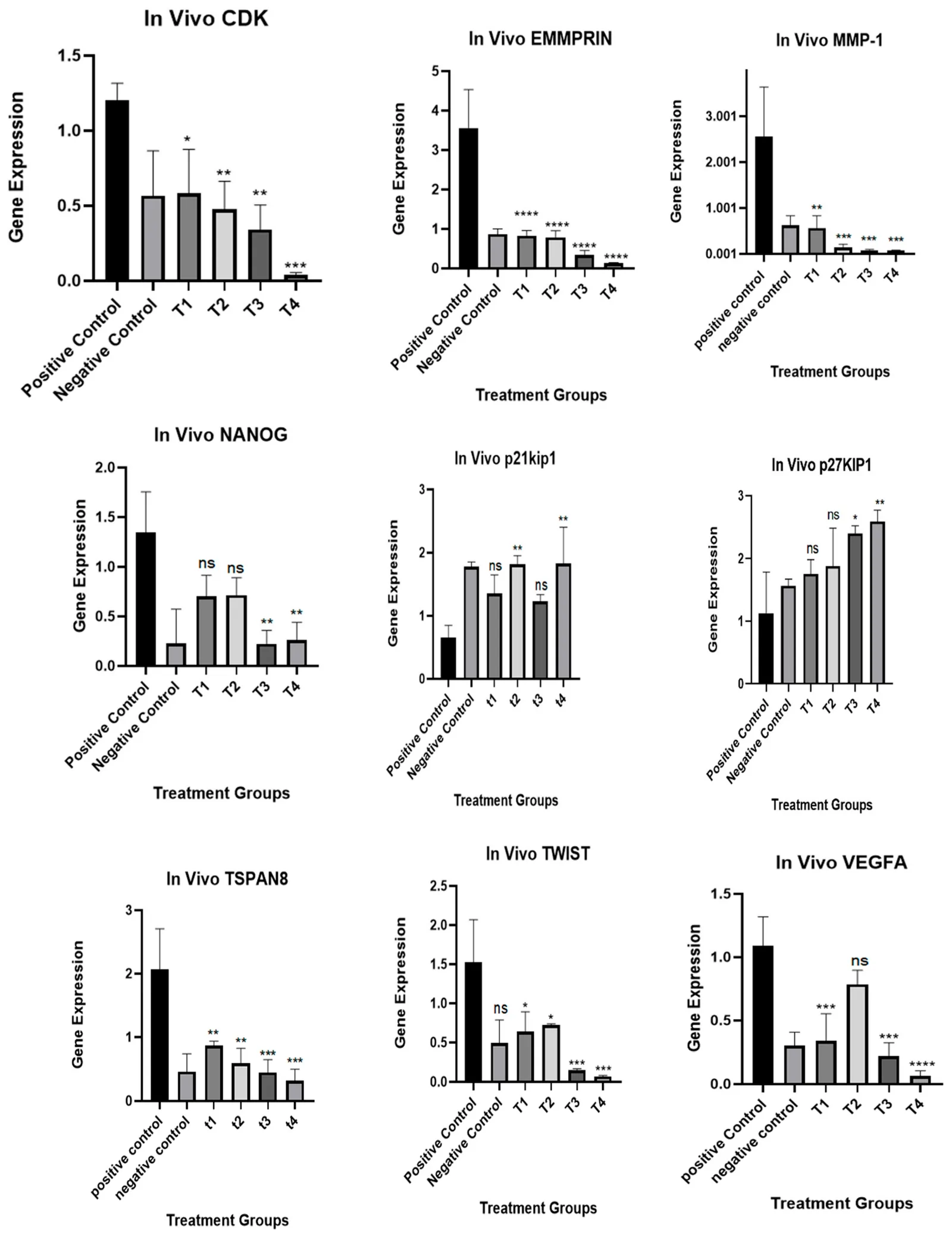
In vivo relative fold changes in gene expression levels within breast tumor tissues across different experimental groups following various treatments. Real-time quantitative PCR (RT-qPCR) analysis targets key regulatory genes involved in cell cycle progression (CDK1, P21^CIP1^, P27^KIP1^), metastasis and extracellular matrix remodeling (EMMPRIN, MMP-1, TSPAN8, TWIST), stemness (NANOG), and angiogenesis (VEGFA). Experimental groups on the *x*-axis are defined as follows: Positive Control (untreated tumor model), Negative Control (healthy model), T1 (Free Coriander), T2 (Coriander loaded on CS NPs), T3 (Free Quercetin [Qu]), T4 (Quercetin loaded on CS NPs), and T5/Negative Control (Free CS NPs, where indicated). Data are presented as mean relative fold expression ± standard deviation (SD). Statistical significance compared to the positive control group is indicated by asterisks: * *p* < 0.05, ** *p* < 0.01, *** *p* < 0.001, **** *p* < 0.0001, while ns denotes a non-significant difference (*p* > 0.05).

The same 9 genes tested out with different treatments for the in vitro study were also analyzed in the in vivo study. In vivo gene expression analysis of the breast tumor samples revealed that all active treatments (T1–T4) significantly modulated key pathways involved in cell cycle progression, angiogenesis, metastasis, and stemness compared to the positive control group. Pro-oncogenic and metastatic markers were consistently downregulated across the treatment groups. Specifically, significant reductions were observed in the expression of the cell cycle regulator *CDK1*, the matrix metalloproteinase inducer *EMMPRIN*, the extracellular matrix remodeling enzyme *MMP-1*, the tetraspanin *TSPAN8*, and the epithelial–mesenchymal transition (EMT) transcription factor *TWIST*. Additionally, the angiogenic driver *VEGFA* and the pluripotency stemness marker *NANOG* were strongly suppressed, with the most profound inhibition of *CDK1* and *VEGFA* recorded in the T4 group (0.35 ± 0.02 fold and 0.06 ± 0.01 fold, respectively), validating the superior efficacy of nanoparticle-delivered quercetin (Figure 10).

Concurrently, the treatments successfully induced tumor-suppressive pathways by upregulating key cell cycle inhibitors. Expression levels of both P21^CIP1^ and P27^KIP1^ were elevated relative to the positive control, with T2 and T4 showing statistically significant increases for P21^CIP1^, while P27^KIP1^ transcription was most effectively activated under free and nanoparticle-loaded quercetin exposure (T3 and T4). Across almost all evaluated genes, the in vivo expression profiles closely mirrored prior in vitro findings, demonstrating that nanoparticle encapsulation—particularly for quercetin (T4)—enhances therapeutic delivery and drives robust anti-tumor, anti-angiogenic, and anti-metastatic effects in tumor tissues.

## 3. Discussion

This study evaluates whether quercetin (Qu) and coriander (Cor) extract—in free form or encapsulated within chitosan-folic acid nanoparticles (CS-FA NPs)—can effectively control breast cancer progression in vitro and in vivo. Direct administration of free phytocompounds often fails due to poor aqueous solubility and rapid systemic clearance. Encapsulation within targeted polymeric carriers successfully bypasses these limitations, enhancing stability and maintaining a sustained therapeutic payload.

Compared with previously reported quercetin-based chitosan nano-formulations, the present work extends current knowledge by integrating folate-mediated targeting with comprehensive biological evaluation across multiple experimental platforms, including mechanistic gene-expression profiling, angiogenesis assessment, and validation in an orthotopic breast cancer model.

The analytical profiling of coriander extract establishes a robust chemical foundation for its therapeutic activity. Gas chromatography–mass spectrometry (GC-MS) and high-performance liquid chromatography (HPLC) confirmed a rich array of phenolic and flavonoid compounds, including caffeic acid, gallic acid, rutin, and a quantifiable quercetin fraction (2.6 mg/g). Because these individual constituents modulate overlapping oncogenic pathways, the natural co-existence of quercetin alongside complementary phenolics within coriander creates potential for synergistic or additive biological activity. Free quercetin independently acts as a pleiotropic agent that downregulates anti-apoptotic proteins, induces cell cycle checkpoints, and suppresses cell proliferation. Consequently, comparing these free agents against their nano-encapsulated counterparts directly isolates the therapeutic value of folate-receptor-mediated drug delivery.

The in vitro findings demonstrate that folate-targeted nano-encapsulation shifts the therapeutic index in favor of selective cytotoxicity. Across MCF-7 (ER+/FR+), MDA-MB-231 (triple-negative, FR+), and 4T1 (murine triple-negative) cell lines, quercetin-loaded nanoparticles (Qu-CS-FA NPs; T4) consistently achieved the most profound reductions in cell viability, outperforming free quercetin (T3) by approximately 20%. This efficacy hierarchy Qu-NP > free Qu > Cor-NP ≈ free Cor ≫ blank NPs underscores the utility of the folate ligand. High-affinity binding to overexpressed folate receptors on malignant cells drives rapid endocytosis of the carrier, whereas normal human skin fibroblasts (HSFs; low FR expression) remain unaffected (>92% viability), a finding supported by broad breast cancer literature regarding quercetin nano-systems. These results align with previous reviews showing that nanoencapsulation significantly enhances quercetin’s antitumor efficacy over its free form, alongside wider evidence demonstrating that folate–chitosan carriers selectively deliver payloads into FR+ breast cancer cells [27,28].

Functional cell motility assays validate this targeted suppression. In scratch assays, T4 treatment almost entirely paralyzed the migratory capacity of aggressive MDA-MB-231 cells, restricting scratch closure to <9% over 48 h. Interestingly, while free quercetin exerted a potent immediate anti-migratory effect, its long-term inhibition waned due to burst release kinetics. In contrast, the nanoparticles exhibited a balanced, biphasic release profile—characterized by an initial rapid delivery (22–26% payload within 10 min) followed by a controlled diffusion phase that plateaued at 66% for quercetin by 8 h. This sustained release maintains therapeutic intracellular drug concentrations, persistently suppressing invasion drivers. This behavior perfectly mirrors observations by [29], who reported that chitosan nanoparticles release quercetin via a biphasic profile where 60–70% displays a rapid burst effect, while the remainder undergoes a sustained 48 h release that prolongs motility inhibition.

Conversely, blank CS-FA NPs and coriander treatments actually facilitated rapid wound closure in healthy models. Chitosan naturally supports structural wound healing by enhancing granulation tissue formation and cell proliferation during repair. Furthermore, coriander contains anti-inflammatory and antimicrobial compounds that accelerate early tissue growth and wound closure in vivo [30], though its capacity to inhibit migration remains strictly cell-type and dosage-dependent [31].

Flow cytometric DNA analysis initially revealed no phase-specific cell cycle arrest (G1, S, or G2/M), seemingly contradicting literature that associates these compounds with defined checkpoint blocks. For instance, ref. [32] demonstrated that *Coriandrum sativum* extracts induce a pronounced G2/M arrest in MCF-7 cells, while quercetin frequently forces a G1 or G2/M arrest by upregulating the CDK inhibitor p21 and modulating p53 and Cyclin D1 [33,34]. However, molecular and phenotypic data resolve this apparent discrepancy. Apoptosis assays demonstrated substantial necrotic and late-apoptotic populations under active treatments, matching evidence that both coriander extracts and quercetin induce extensive programmed cell death via mitochondrial and death-receptor pathways [35,36]. This dominant cytotoxic stress induces rapid cell death and extensive DNA fragmentation, which interferes with propidium iodide staining and masks classic cell-cycle distribution patterns [37].

Crucially, real-time quantitative PCR (RT-qPCR) provided definitive evidence that cell cycle inhibitory pathways were fully activated at the transcript level. Active treatments strongly upregulated the cyclin-dependent kinase inhibitors P21^CIP1^ and P27^KIP1^, which are known to deactivate CDK2 Cyclin E complexes and prime cells for growth arrest [38]. Because gene expression alterations precede physical phenotypic shifts, the rapid onset of apoptosis simply overtook the cells before a static, phase-specific block could register via flow cytometry under conditions of high cytotoxic stress [39]. Rather than acting through cytostatic delays, these nano-formulations force a definitive exit from the cell cycle directly into programmed cell death.

Metabolic reprogramming is now recognized as a core driver of breast-cancer aggressiveness. Ref. [40] showed that the oncogene MAEL promotes aerobic glycolysis by chaperone-mediated autophagy-dependent degradation of the TCA-cycle enzymes citrate synthase and fumarate hydratase, thereby fueling malignant progression. Although our study did not directly measure glycolytic flux or TCA metabolites, the transcriptional changes we recorded—markedly reduced expression of the stemness factor NANOG, the mitotic kinase CDK1, the angiogenic driver VEGFA and several EMT/invasion genes—point to a parallel collapse of the networks that sustain the glycolytic, proliferative and metastatic phenotype. In particular, the folate-targeted quercetin nanoparticles (T4) produced the most consistent multi-gene repression both in vitro and in the orthotopic 4T1 model. These observations suggest that improved intracellular delivery of quercetin (and the complementary phenolics present in coriander extract) can interrupt several of the same downstream programs that MAEL-driven metabolic rewiring supports, offering a pharmacological route to reprogram the tumor cell state without directly targeting MAEL itself.

Functional ex vivo and in vivo evaluations confirm that these formulations disrupt the broader physiological networks required for tumor survival. In the ex vivo rat aortic ring assay, T4 mediated near-complete abolition of microvessel sprouting. This functional anti-angiogenic response correlates directly with a dramatic, 94% downregulation of vascular endothelial growth factor A (*VEGFA*) expression in aggressive tumor models. By restricting *VEGFA* transcription—likely through upstream inhibition of the PI3K/Akt, MAPK, and HIF-1⍺ axes—the nanoparticle platform successfully starves the tumor of necessary vascular recruitment [41,42].

VEGFA is a key regulator of angiogenesis that promotes endothelial cell proliferation, migration, and survival while facilitating vascular remodeling necessary for sustained tumor growth and metastatic dissemination. Consequently, suppression of VEGFA expression disrupts the formation of functional tumor vasculature, limiting oxygen and nutrient delivery to rapidly proliferating cancer cells. The marked inhibition of microvessel sprouting observed in the rat aortic ring assay is therefore consistent with the molecular findings and provides functional evidence that folate-functionalized quercetin-loaded chitosan nanoparticles effectively suppress angiogenic signaling through coordinated inhibition of VEGFA-driven pathways.

In the in vivo 4T1 murine breast cancer model, untreated positive controls displayed an aggressive malignant phenotype, characterized by heavily elevated serum tumor markers CA15-3 at 35 U/mL and arginase at 366 U/L), high mitotic indices, and pleomorphic spindle-shaped structures. These parameters reflect a classic advanced breast cancer signature, where CA15-3 tracks tumor burden, and arginase is upregulated to facilitate polyamine synthesis and establish an immunosuppressive microenvironment [43,44,45]. Systemic treatment with Qu-CS-FA NPs (T4) reversed this malignant signature by orchestrating a comprehensive, multi-genic shutdown of oncogenic pathways: Proliferation and Survival: Rebalanced cell cycle controls by significantly downregulating the G2/M driver *CDK1* while upregulating *P21* and *P27* to enforce arrest and apoptosis [46,47,48], Stemness: Suppressed the pluripotency transcription factor *NANOG* by over 90%, effectively reducing the “stem-like” self-renewal signals that drive tumor recurrence and chemoresistance [49], and invasion and metastasis: Severely repressed the metastatic cascade. The extracellular matrix remodeling enzyme *MMP-1* was nearly silenced, its upstream inducer *EMMPRIN* was cut to 0.33 times control levels, and the tetraspanin *TSPAN8* was heavily down-regulated [47,50]. Furthermore, the master epithelial–mesenchymal transition (EMT) transcription factor *TWIST1* was reduced to just 14% of control levels. This profound suppression confirms a potent anti-EMT effect, matching evidence that quercetin directly counteracts metastasis by blocking transcription factors like TWIST, SNAIL, and downstream matrix metalloproteinases [9,51,52,53].

This study demonstrates that while free quercetin and coriander extract possess inherent anticancer bioactivity, their therapeutic potential is strictly limited by physiological delivery barriers. Encapsulation within chitosan-folic acid nanoparticles successfully resolves these limitations. By exploiting optimal physical dimensions (a 150 nm hydrodynamic size, an encapsulation efficiency of approximately 80 to 90%, and a −6 to −15 mV zeta potential and an active folate-targeting ligand, the nano-formulations maximize intracellular payload delivery via receptor-mediated endocytosis [27,28].

The higher encapsulation efficiency and drug loading observed for quercetin compared with the coriander extract are likely related to its physicochemical properties, including its relatively hydrophobic nature and favorable interaction with the chitosan matrix. In contrast, the crude coriander extract contains multiple phytochemical constituents with different molecular sizes and polarities, which may reduce loading efficiency due to competitive interactions during adsorption.

The preserved spherical morphology together with the narrow particle size distribution suggests that adsorption of quercetin and *Coriandrum sativum* extract, followed by folate surface functionalization, did not noticeably compromise the structural integrity of the chitosan nanoparticles. Such morphology is considered advantageous for cellular uptake and intracellular drug delivery while the combination of a hydrodynamic diameter within the nanometer range and a highly negative zeta potential indicates excellent colloidal stability, reducing particle aggregation through electrostatic repulsion. These physicochemical characteristics are particularly important for maintaining nanoparticle dispersion during biological applications and facilitating reproducible cellular interactions.

Ultimately, quercetin-loaded nanoparticles (T4) represent the most effective strategy, demonstrating a clear capacity to control breast cancer progression in vitro and in vivo through simultaneous cell-cycle deactivation, rapid apoptotic induction, and the multi-target suppression of angiogenesis, stemness, and EMT pathways.

## 4. Materials and Methods

### 4.1. Plant Material and Reference Standards

*Coriandrum sativum* (coriander) seeds (99% purity, 5% moisture, machine-dried at 8% humidity) were procured from Alfa Herbs (Cairo, Egypt). Analytical standard quercetin vials (>98% HPLC purity) were obtained from Nawah Scientific Inc. (Mokatam, Cairo, Egypt).

### 4.2. Nanoparticle Synthesis Reagents

High molecular weight chitosan, glacial acetic acid (GAA), Tween 80, folic acid, Dimethyl Sulfoxide (DMSO), methanol (HPLC grade), and distilled water were purchased from Piochem (Giza, Egypt). Sodium tripolyphosphate (STPP) was acquired from Lab Chemical Trading Co. (Qasr El Einy, Egypt).

### 4.3. Cell Lines and Biological Reagents

Human breast adenocarcinoma (MCF-7), triple-negative breast cancer cells (MDA-MB-231), mouse breast cancer cells (4T-1), and human skin fibroblasts (HSFs) were obtained from Nawah Scientific Inc. (Mokatam, Cairo, Egypt). Cell culture consumables, including Dulbecco’s Modified Eagle Medium (DMEM), phosphate-buffered saline (PBS), fetal bovine serum (FBS), penicillin-streptomycin, L-glutamine, trypsin-EDTA, and TRIzol were purchased from Thermo Fisher Scientific. Endothelial Basal Medium (EBM) was sourced from Lonza. Rat tail collagen type I, MTT solution, solubilization solution, and sterile 10× minimum essential medium (MEM) were obtained from Sigma-Aldrich (Taufkirchen, Germany).

### 4.4. Molecular Biology Kits

The Total RNA Purification Kit was obtained from Norgen Biotek Corp (Thorold, ON, Canada). RevertAid First Strand cDNA Synthesis Kit and DNase I (RNase-free) kit were purchased from Thermo Fisher Scientific Inc. (Waltham, MA, USA) The HERA SYBR^®^ Green qPCR Kit was sourced from Willowfort Co. (Birmingham, UK), and the FavorPrep™ Tissue Total RNA Mini Kit was obtained from Favorgen Biotech Corp. (Pingtung, Taiwan).

### 4.5. Phytochemical Analysis

*Extraction*: Powdered *C. sativum* seeds were extracted in 80% methanol (1:4 *w*/*v*) at room temperature for 72 h under intermittent agitation, filtered via Whatman No. 1 paper, and concentrated using a rotary evaporator (Heidolph, Germany) at 40 °C to yield a semi-solid crude extract.

*GC-MS Profiling*: Volatile profiling was performed on a Gas Chromatography–Mass Spectrometry system (ISQ 7000, Thermo Scientific™) utilizing Xcalibur software (version 4.3). Separation was carried out on an Rxi-5Sil MS capillary column (30 m × 0.25 mm ID, 0.25 µm film thickness) with helium carrier gas (1.0 mL/min). The injector was set to 250 °C with a temperature program spanning 50 °C to 280 °C (5 °C/min). Compound identification was achieved by spectral matching against the NIST Mass Spectral Library.

*HPLC-UV Quantification*: Flavonoid and phenolic quantification was executed on an Agilent 1260 Infinity HPLC system equipped with a UV detector. Chromatography was managed using a C18 reversed-phase column (250 mm × 4.6 mm, 5 µm) at ambient temperature. The mobile phase comprised methanol (A) and 0.1% formic acid in water (B) running a gradient of 20% to 80% A over 30 min at 1.0 mL/min. Detection was fixed at 370 nm, calibrated against a standard curve of quercetin (25–400 ppm).

### 4.6. Formulation and Characterization of Chitosan Nanoparticles (CS NPs)

#### 4.6.1. Synthesis and Bioactive Loading

Folic acid-functionalized chitosan nanoparticles (CS NPs) were synthesized via a modified ionic gelation technique using a 0.25% *w*/*v* chitosan solution in 0.5% *v*/*v* aqueous acetic acid, cross-linked through the dropwise addition of 1% *w*/*v* sodium tripolyphosphate (STPP) under continuous magnetic stirring. Surface adsorption of bioactive components was accomplished by mixing the developed CS NPs suspension for 2 h with either standard quercetin or *C. sativum* crude extract dissolved in a Tween 80/PBS vehicle. Formulations were isolated via centrifugation at 8000 rpm for 10 min and washed with sterile saline or PBS.

The formulation conditions were selected based on preliminary optimization experiments and previously reported chitosan nanoparticle preparation protocols to obtain nanoparticles with suitable particle size, colloidal stability, and encapsulation efficiency. The selected chitosan, TPP, and Tween 80 concentrations consistently produced homogeneous nanoparticles appropriate for subsequent biological evaluation.

Following nanoparticle formation, folic acid was introduced as a surface-functionalizing ligand to enhance tumor-targeting capability. Surface modification was achieved by allowing folic acid to adsorb onto the chitosan nanoparticle surface through electrostatic interactions and hydrogen bonding between the negatively charged carboxyl groups of folic acid and the positively charged amino groups of chitosan. Performing this step after ionic gelation ensured that folate molecules remained exposed on the nanoparticle surface, thereby facilitating folate receptor-mediated cellular uptake while minimizing interference with nanoparticle formation.

#### 4.6.2. Physicochemical Characterization

Morphological properties and particle size were evaluated using a JEM-2100 high-resolution transmission electron microscope (JEOL, Tokyo, Japan). Hydrodynamic dimensions, size distribution, and surface charge metrics were monitored via Dynamic Light Scattering (DLS) using a Zetasizer Nano ZS system (Malvern Instruments Ltd., Malvern, UK). Functional groups were analyzed by a JASCO 6700 Fourier transform infrared (FT-IR) spectrometer (JASCO Corporation, Tokyo, Japan).

### 4.7. In Vitro Drug Release Kinetics

Cumulative release kinetics were determined using a USP Type II paddle dissolution apparatus (PG Instruments, Lutterworth, UK) operating at 37 ± 0.5 °C in 900 mL of dissolution medium at 50–100 rpm. Aliquots sampled at specified intervals up to 480 min were analyzed via UV-Vis spectrophotometry (ƛ-max). Mathematical profiles were fitted into Zero-order, First-order, Higuchi, and Hixson–Crowell kinetic models to confirm release mechanisms.

### 4.8. In Vitro Biological Assays

#### 4.8.1. Cell Culture and MTT Cytotoxicity Assay

Cell lines were maintained in DMEM supplemented with 10% heat-inactivated FBS, 100 units/mL penicillin, and 100 mg/mL streptomycin at 37 °C under a humidified 5% CO_2_ atmosphere. Metabolic viability across treated configurations (free extract, free quercetin, extract-loaded CS NPs, quercetin-loaded CS NPs, and blank CS NPs) was analyzed using standard MTT protocols, measuring differential absorbance profiles through a microplate reader at 450 nm and 630 nm.

#### 4.8.2. Wound Healing Scratch Assay

Cell migration behaviors were quantified by scratching confluent monolayers with fine pipette tips. Scratched boundaries were continuously examined under a phase-contrast microscope across 0, 24, and 48 h windows to compute migration rates (Rm) and wound closure percentages.

#### 4.8.3. Flow Cytometric Analysis of Cell Cycle and Apoptosis

Cells were treated with each formulation at their respective IC50 concentration, as determined by the MTT cytotoxicity assay, and incubated for the specified duration prior to apoptosis analysis. For cell cycle tracking, fixed cells (60% ice-cold ethanol) were stained with propidium iodide (PI, 10 µg/mL) containing RNAase A (50 µg/mL) and quantified on an ACEA Novocyte™ flow cytometer (ACEA Biosciences Inc., San Diego, CA, USA). Apoptosis profiling was conducted utilizing an Annexin V-FITC Apoptosis Detection Kit (Abcam Inc., Cambridge, UK) following quadrant-based fluorescence processing on ACEA NovoExpress™ software, version 1.1.0. Quadrant analysis was used to classify cells as viable (Annexin V−/PI−), early apoptotic (Annexin V+/PI−), late apoptotic (Annexin V+/PI+), and necrotic (Annexin V−/PI+) according to the manufacturer’s protocol.

### 4.9. Angiogenic Aortic Ring Assay

Ex vivo vascular sprouting was monitored utilizing rat aortic rings embedded in a localized type I collagen matrix generated using 10× MEM, sodium bicarbonate, and collagen solution. Ring setups were co-cultured with or without adjacent cancer cell aggregate matrices in serum-free EBM. Microscopic evaluation of endothelial capillary sprouting tracks was performed via an inverted bright-field microscope.

### 4.10. Quantitative Real-Time PCR (qPCR) Gene Expression

Total RNA isolation from in vitro cells and in vivo mammary tissue blocks was executed using the Norgen Biotek RNA Purification Kit and FavorPrep™ Tissue Total RNA Mini Kit, respectively. Isolated transcripts underwent genomic DNA digestion via the DNase I, RNase-free kit (Thermo Fisher Scientific). Reverse transcription was carried out using the RevertAid First Strand cDNA Synthesis Kit (Thermo Fisher Scientific).

Real-time qPCR amplification was implemented on a 96-well validation setup incorporating the HERAPLUS SYBR^®^ Green qPCR Kit (Willowfort Co., Nottingham, UK) targeting critical regulatory markers (Nanog, TSPAN8, CDK1, EMMPRIN, P21^CIP1^, P27^KIP1^, MMP1, TWIST, and VEGFA), normalized against house-keeping GAPDH expressions. Primers used in this study are presented in Table 5.

### 4.11. In Vivo Animal Models and Experimental Design

#### 4.11.1. Ethical Statement and Environmental Conditions

Fifty female Balb/c mice were procured from Cairo University (Giza, Egypt). All experimental workflows were conducted at the University Research Animal Facility (URAF) in accordance with the institutional guidelines for the care and use of laboratory animals. The study protocol was reviewed and formally sanctioned by the Institutional Animal Care and Use Committee (IACUC) of Cairo University. The animals were maintained in a specific-pathogen-free (SPF) environment under a regulated 12 h light/dark cycle and a stable ambient temperature of 22 ± 2 °C, with ad libitum access to standard laboratory chow and water.

#### 4.11.2. Orthotopic Tumor Induction

Following a 7-day acclimation period, syngeneic mammary tumors were orthotopically induced in the mice. Briefly, log-phase 4T1 mouse breast cancer cells were harvested, washed, and resuspended in sterile phosphate-buffered saline (PBS). An aliquot of 100 µL containing 1 × 10^5^ cells was inoculated via intramammary injection directly into the fourth pair of abdominal mammary glands. Following inoculation, neoplastic progression was actively monitored over a 10-day period. Perpendicular tumor dimensions were recorded every 2 to 3 days using electronic calipers, and the total tumor burden was expressed as the tumor area (mm^2^) derived from the product of the two bidirectional measurements. Animals displaying palpable mass formations were selected for subsequent therapeutic interventions.

#### 4.11.3. Experimental Stratification and Therapeutic Regimen

Tumor-bearing mice were randomly assigned to the experimental groups after successful tumor establishment to minimize allocation bias. (n = 8 per group) as follows: Group 1 (Negative Control): Healthy mice receiving a matching 100 µL intramammary injection of sterile PBS, Group 2 (Positive Control): Tumor-bearing mice receiving 100 µL intramammary injections of 4T1 cells without therapeutic intervention, Group 3: Tumor-bearing mice treated with free quercetin, Group 4: Tumor-bearing mice treated with quercetin-loaded chitosan nanoparticles (Qu-CS NPs), Group 5: Tumor-bearing mice treated with free *Coriandrum sativum* (coriander) crude extract, and Group 6: Tumor-bearing mice treated with coriander crude extract-loaded chitosan nanoparticles (coriander-CS NPs). Allocation was performed using the order of animals within the cages to distribute animals sequentially among the treatment groups.

A total of eight animals per group was selected based on previous comparable breast cancer studies using the 4T1 murine model and practical considerations regarding ethical animal use. No formal priori sample size calculation was performed.

All active therapeutic arms (Groups 3–6) received a standardized daily dose of 50 mg/kg of their respective formulations over a continuous period of 14 days.

No animals were excluded after randomization, and all animals completed the study according to the predefined experimental protocol.

#### 4.11.4. Terminal Specimen Assembly and Processing

Upon completion of the 14-day treatment window, the mice were anesthetized via an intraperitoneal injection of ketamine (80 mg) and xylazine (8 mg). Whole blood samples were collected via the retro-orbital sinus using EDTA microtubes to isolate plasma fractions via centrifugation (3000 RPM at 4 °C for 10 min) for downstream CA15-3 tumor marker and arginase biochemical profiling. Following blood collection, the animals were humanely euthanized.

Animals were monitored daily throughout the experiment for general health status and signs of distress. No animals reached predefined humane endpoints requiring early euthanasia before the planned study termination.

The target mammary glands and associated tumor tissues were rapidly excised and thoroughly rinsed with ice-cold physiological saline to eliminate lingering blood traces. Collected tissue blocks were partitioned for distinct analytical endpoints: One fraction was immediately submerged in 10% formal saline (or neutral buffered formalin) to fix cellular structures for subsequent histopathological analysis. The remaining tissue portions were instantly immersed in TRIzol lysis reagent and transferred to ultra-low temperature storage at −70 °C to preserve transcript integrity for real-time quantitative PCR (qPCR) gene expression analysis.

### 4.12. Serum Marker and Arginase Assays

Circulating breast cancer progress index marks (CA15-3) and relative plasma arginase activity levels—quantified via enzyme-catalyzed urea conversion configurations from arginine—were assessed from separated blood fractions collected during terminal processing.

### 4.13. Histopathological Examination

Excised mammary gland tissue sections were fixed in 10% neutral buffered formalin, routinely processed through automated ascending ethanol-xylene dehydration paths, and embedded in paraffin wax. Fine tissue sections (3–5 µm) cut via a rotary microtome were stained with Hematoxylin and Eosin (H&E) to characterize tissue architecture, cellular features, and mitotic indicators under a light microscope. Blinding was not implemented during outcome assessment because the treatments were administered and analyzed by the same research team.

No unexpected adverse events related to the administered treatments were observed during the experimental period.

### 4.14. Statistical Analysis

Quantitative experimental data are presented as mean ± standard deviation (SD) from at least three independent biological replicates to confirm reproducibility. Statistical evaluations and comparative analyses between the experimental and control cohorts were executed using one-way analysis of variance (ANOVA), followed by Tukey’s post hoc multiple comparison tests for inter-group regularities. For differential gene expression profiling, statistical significance between specific treatment matrices and corresponding controls was determined using an independent Student’s *t*-test. In all biological and molecular evaluations, a *p*-value of less than 0.05 (*p* < 0.05) was strictly maintained as the threshold for statistical significance. All statistical computations were processed to ensure robust validation and accurate interpretation of the therapeutic outcomes.

## 5. Conclusions

In conclusion, this investigation successfully demonstrated that the encapsulation of standard quercetin and *Coriandrum sativum* seed extract within folate-functionalized chitosan nanoparticles significantly overcomes the biopharmaceutical limitations of free natural bioactives, establishing a potent, multi-targeted platform for breast cancer oncotherapy. The engineered CS-FA polymeric nanocarriers exhibited excellent physicochemical features, high encapsulation capacity, exceptional colloidal stability, and a controlled, diffusion-driven release profile. Biologically, the folate-ligand modification provided target-specific delivery that selectively spared healthy fibroblasts while inducing significant, cell-line-dependent cytotoxicity across luminal and triple-negative breast cancer lines. This therapeutic response was driven by a dual-axis molecular mechanism: the induction of catastrophic cellular apoptosis and necrosis, and the suppression of the tumor microenvironment through ex vivo anti-angiogenic vascular collapse. At the transcriptomic level, the nano-formulations acted as robust key regulators, shifting the expression of critical regulatory genes to simultaneously enforce mitotic block, silence stemness, and dismantle the epithelial–mesenchymal transition and metastatic cascade. Ultimately, the in vivo validation using a syngeneic orthotopic model confirmed the translational potential of these nano-formulations, as evidenced by the dramatic reduction in circulating clinical tumor markers, the suppression of immunosuppressive arginase activity, and the successful structural restoration of malignant mammary tissues.

**Future Perspectives**

The encouraging therapeutic performance of the folate-functionalized chitosan nano-formulations highlights several opportunities for future development. Further optimization may include multifunctional nanoparticle systems capable of co-delivering phytochemicals with conventional chemotherapeutic agents or immunotherapeutic drugs to achieve synergistic anticancer effects. Incorporation of stimuli-responsive drug release mechanisms, such as pH- or enzyme-sensitive delivery systems, may further improve tumor-selective drug release while minimizing systemic toxicity. In addition, integrating diagnostic and therapeutic functions into a single theranostic nanoplatform could enable simultaneous tumor imaging and targeted treatment. Advances in artificial intelligence and machine-learning approaches also offer promising opportunities for optimizing nanoparticle formulation parameters and predicting biological performance. Ultimately, combining targeted nanomedicine with patient-specific molecular profiling may facilitate the development of personalized therapeutic strategies that maximize efficacy while reducing adverse effects in breast cancer patients.

## Figures and Tables

**Table 1:** Major constituents found in Coriander.

Retention Time (min)	Compound Name	Area %	Molecular Formula	Remarks
9.202	*n*-Hexadecanoic acid (Palmitic acid)	13.77	C_16_H_32_O_2_	Fatty acid; antimicrobial, antioxidant
11.120	Phytol	7.66	C_20_H_40_O	Antimicrobial, vitamin precursor
7.213	Cis-vaccenic acid	5.34	C_18_H_34_O_2_	Unsaturated fatty acid
15.001	9,12-Octadecadienoic acid (Linoleic)	3.89	C_18_H_32_O_2_	Antioxidant, anti-cancer activity
12.921	Quercetin	2.72	C_15_H_10_O_7_	Major flavonoid; key bioactive compound
10.351	Stigmasterol	1.95	C_29_H_48_O	Sterol; cholesterol-lowering effects

**Table 2:** Phenolic and Flavonoid Compounds in the methanolic extract of *Coriander Sativum*.

Retention Time (min)	Compound	λmax (nm)	Conc. (mg/g Extract)	Peak Area (%)	Biological Role
3.2	Gallic acid	272	1.2	4.1%	Phenolic acid; antioxidant
4.7	Caffeic acid	325	1.0	3.5%	Hydroxycinnamic acid
6.1	Quercetin	370	2.6	8.5%	Major flavonoid; antioxidant
7.8	Luteolin	350	1.1	3.7%	Flavone; anti-inflammatory
9.3	Kaempferol	365	0.8	2.9%	Flavonoid; anticancer activity
—	Unknown flavonoids	—	~1.5	~5.0%	Uncharacterized bioactives

**Table 3:** Mathematical model fitting and coefficient of determination (R^2^) for the release kinetics of quercetin- and coriander-loaded chitosan-folic acid nanoparticles (CS-FA-NPs).

Model	Quercetin-CS-FA-NPs (R^2^)	Best Fit	Coriander-CS-FA-NPs (R^2^)	Best Fit
Zero-order	0.105	No	0.283	No
First-order	0.003	No	0.097	No
Higuchi	0.241	Yes	0.481	Yes
Hixson–Crowell	0.023	No	0.180	No

**Table 4:** MTT cell-viability after 48 h single-dose exposure in breast cancer (MCF-7, MDA-MB-231, 4T1) and normal fibroblast (HSF) cell lines.

Cell Line	Control	T1 (Free Coriander)	T2 (Cor-CS-FA-NPs)	T3 (Free Quercetin)	T4 (Qu-CS-FA-NPs)	T5 (Blank NPs)
MCF-7	100%	82 ± 4%	70 ± 3%	65 ± 3%	45 ± 2%	95 ± 3%
MDA-MB-231	100%	85 ± 5%	72 ± 4%	60 ± 3%	40 ± 2%	96 ± 3%
4T1	100%	88 ± 4%	75 ± 3%	62 ± 4%	42 ± 3%	94 ± 2%
HSF	100%	96 ± 2%	95 ± 2%	94 ± 3%	92 ± 3%	98 ± 2%

**Table 5:** Oligonucleotide Primer Sequences Utilized for Quantitative Real-Time PCR (RT-qPCR).

Gene	Forward Primer (5′ → 3′)	Reverse Primer (5′ → 3′)
***Nanog***	CCTCAGCACCTACCTACCCC	AAGGTTCCCAGTCGGGTTCA
***TSPAN8***	AGGTGGCGACAGGTATCCTA	TTTCACTTTCCCCTGTGGCG
***CDK1***	ACTGGCTGATTTTGGCCTTG	GAGCTGACCCCAGCAATACT
***EMMPRIN***	GAGGACACGGGCACTTACG	GTGAAGACTGTGCCGGGTT
***P21^CIP1^***	CGGGTGCGGTGATGGATAAA	GGAAGAACTCGGGGAACGTC
***P27^KIP1^***	TAATTGGGGCTCCGGCTAAC	TTGGGGAACCGTCTGAAACA
***MMP1***	CACAGCTTTCCTCCACTGCT	GCCTCCCATCATTCTTCAGGT
***TWIST***	TCAAAGAAACAGGGCGTGGG	CCGTCTGGGAATCACTGTCC
***VEGFA***	GGGAAAGGGGCAAAAACGAAA	CATTAGACAGCAGCGGGCA
***GAPDH***	TAGGCGCTCACTGTTCTCTC	GCCCAATACGACCAAATCCG

## Data Availability

The original contributions presented in this study are included in the article and Supplementary Materials. Further inquiries can be directed to the corresponding author.

## Supplementary Materials

The following supporting information can be downloaded at: https://www.mdpi.com/article/10.3390/ph19081271/s1. Table S1: Complete GC-MS profile of the methanolic extract of Coriandrum sativum seeds.
