# Supplementary material for: Targeted Folate-Chitosan Nanoformulations of Quercetin and Coriandrum sativum Reprogram Breast Cancer Hallmarks by Silencing Stemness, Cell Cycle, Angiogenic, and Metastatic Networks
Source: Pharmaceuticals (Basel). 2026 Aug 12;19(8):1271. doi: 10.3390/ph19081271 (PMC13516653; doi:10.3390/ph19081271)
Supplement: Supplementary file 1 [file pharmaceuticals-19-01271-s001.zip › pharmaceuticals-4436947-supplementary.pdf]

Supplementary Table S1. Complete GC-MS profile of the methanolic extract of Coriandrum sativum seeds.

RT (min)	Area %	Peak Area	Peak Height	Compound Name (Top Library Match)	Prob. (%)	MW	Molecular Formula
5.04	2.18	759534	229757	(Unidentified / minor)	-	-	-
6.63	2.72	946736	165067	5,11,17,23-Tetra-t-butyl-...-cyclo-d-arene	68.74	648	$C_{44}H_{56}O_4$
6.81	5.30	1848017	293616	Acetamide, N-[2-(acetyloxy)-2-[4-(acetyloxy)phenyl]ethyl]-	14.96	279	$C_{14}H_{17}NO_5$
6.99	3.95	1376990	231564	(Mixed / minor)	-	-	-
7.10	1.29	448280	145430	Ethanol, 2-(9,12-octadecadienyloxy)-...	~6	310	$C_{20}H_{38}O_2$
7.17	1.21	423119	136163	(Silane / minor derivative)	-	-	-
8.42	0.95	332288	65094	(Minor)	-	-	-
10.11	1.64	573378	85636	(Minor)	-	-	-
11.52	1.03	359769	64490	(Minor)	-	-	-
12.11	1.06	371006	53147	(Minor)	-	-	-
12.921	~2.72	-	-	**Quercetin** (key bioactive)	-	302	$C_{15}H_{10}O_7$
13.98	1.09	380658	66105	Ceanothine C	25.12	470	$C_{26}H_{38}N_4O_4$
15.001	~3.89	-	-	9,12-Octadecadienoic acid (Linoleic acid)	-	280	$C_{18}H_{32}O_2$
16.09	1.45	504011	62421	(Minor)	-	-	-
16.44	2.09	727264	131171	(Minor)	-	-	-
16.56	1.04	361700	-	Hexadecatrienoic acid, methyl ester	~6	264	$C_{17}H_{28}O_2$
17.18–17.67	~1.1–1.3	~387k–449k	~108k–110k	(Multiple minor)	-	-	-
18.79	1.23	429524	138898	9-Hexadecenoic acid, 9-hexadecenyl ester	-	478	$C_{32}H_{62}O_2$
19.202	13.77	-	-	**n-Hexadecanoic acid (Palmitic acid)**	-	256	$C_{16}H_{32}O_2$
20.52–21.66	~1.1–2.1	~392k–740k	~89k–126k	(Fatty acid esters / minor)	-	-	-
7.213	5.34	-	-	**Cis-vaccenic acid**	-	282	$C_{18}H_{34}O_2$
11.120	7.66	-	-	**Phytol**	-	296	$C_{20}H_{40}O$
35.67	8.85	3086054	468319	(Major unresolved peak)	-	-	-
39.04	18.28	6370817	790627	**Dominant peak (likely mixture)**	-	-	-
46.13	0.99	344531	74573	Eicosane / 8-Octadecenal	~7	282	$C_{20}H_{42}$ / $C_{18}H_{34}O$
(remaining minor peaks up to 53.84)	0.95–1.67	-	-	Various fatty acids, terpenoids, sterols (full list available in raw report)	-	-	-
